# Beyond Strain Release: Delocalization-Enabled Organic Reactivity

**DOI:** 10.1021/acs.joc.4c00857

**Published:** 2024-07-06

**Authors:** Alistair J. Sterling, Russell C. Smith, Edward A. Anderson, Fernanda Duarte

**Affiliations:** † Chemistry Research Laboratory, University of Oxford, 12 Mansfield Road, Oxford OX1 3TA, U.K.; ‡ Department of Chemistry & Biochemistry, The University of Texas at Dallas, 800 W. Campbell Rad, Richardson, Texas 75080, United States; § Abbvie Drug Discovery Science & Technology (DDST), 1 North Waukegan Road, North Chicago, Illinois 60064, United States

## Abstract

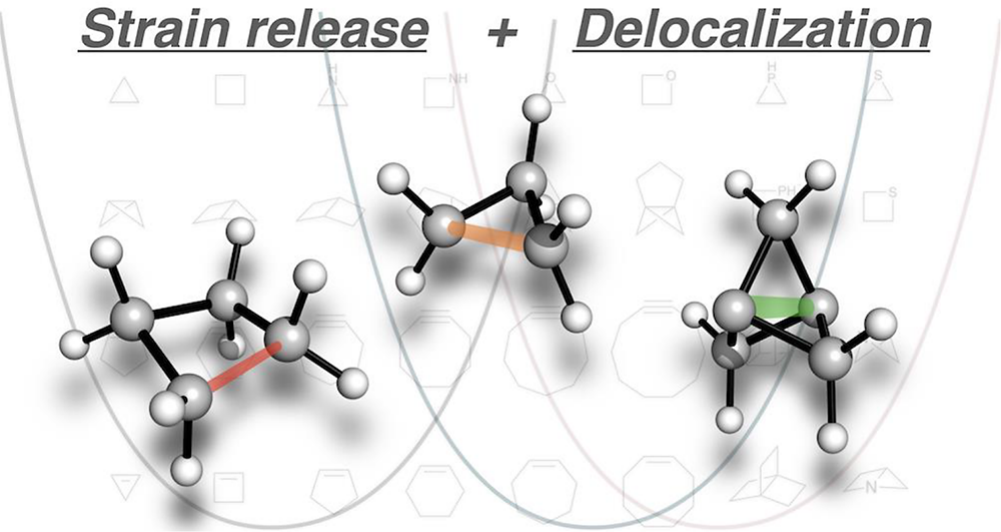

The release of strain energy is a fundamental driving force for organic reactions. However, absolute strain energy alone is an insufficient predictor of reactivity, evidenced by the similar ring strain but disparate reactivity of cyclopropanes and cyclobutanes. In this work, we demonstrate that electronic delocalization is a key factor that operates alongside strain release to boost, or even dominate, reactivity. This delocalization principle extends across a wide range of molecules containing three-membered rings such as epoxides, aziridines, and propellanes and also applies to strain-driven cycloaddition reactions. Our findings lead to a “rule of thumb” for the accurate prediction of activation barriers in such systems, which can be easily applied to reactions involving many of the strained building blocks commonly encountered in organic synthesis, medicinal chemistry, polymer science, and bioconjugation. Given the significance of electronic delocalization in organic chemistry, for example in aromatic π-systems and hyperconjugation, we anticipate that this concept will serve as a versatile tool to understand and predict organic reactivity.

## Introduction

**Figure 1:**
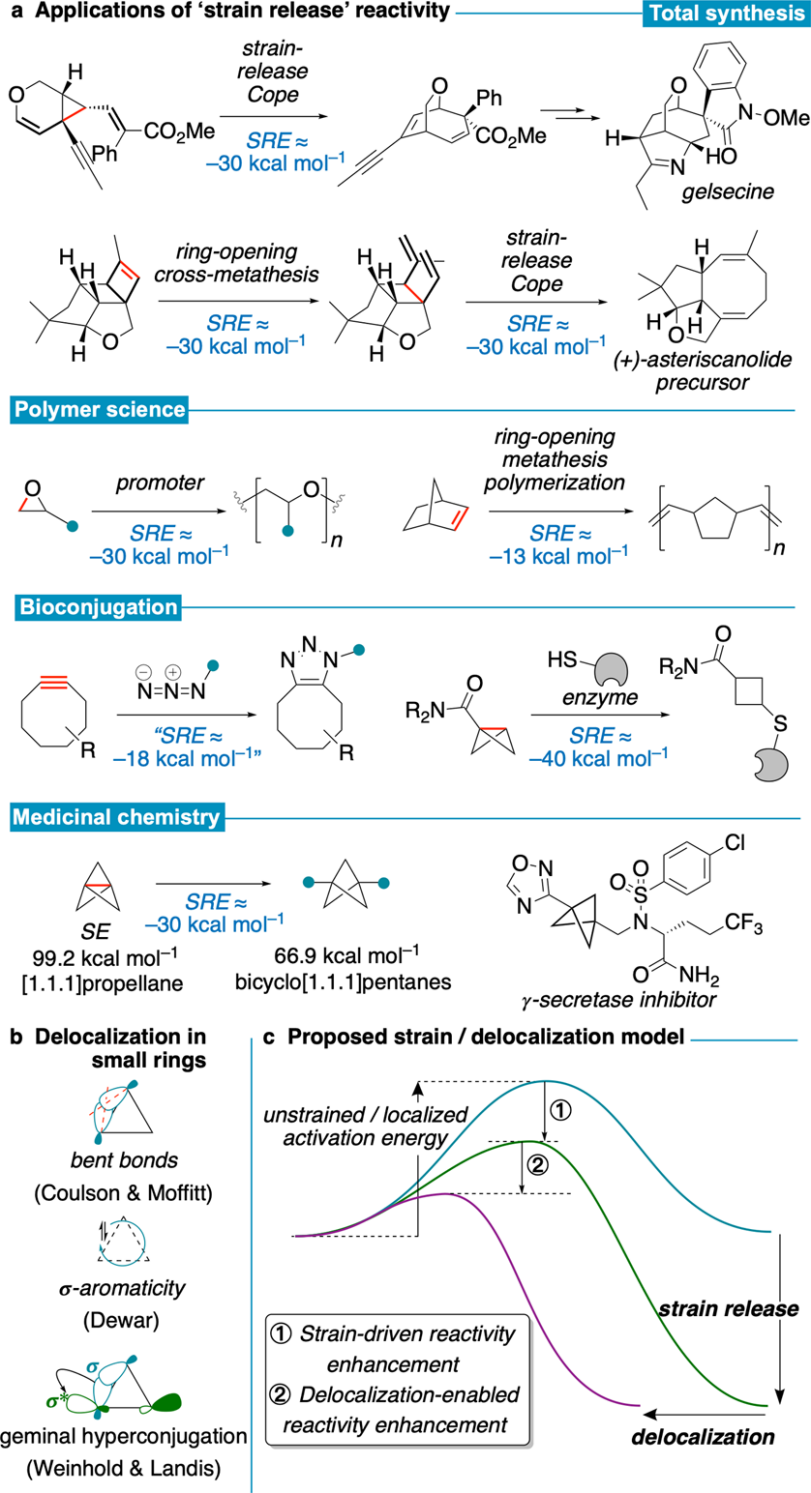
Ring strain in organic chemistry. (a) Examples of strain release-driven reactivity, including total synthesis,^16,17^ bioconjugation reactions,^7,8^ ring-opening polymerization,^18^ and bioisostere synthesis.^19^ (b) Ground state models for electron delocalization in three-membered rings. (c) This work: strain release and delocalization combine to enhance reactivity through lower activation barriers and earlier TSs.

The release of molecular strain has long been harnessed as a powerful driving force in chemical synthesis. A fundamental concept in organic chemistry is “ring strain”,^1,2^ which is used to explain the heightened reactivity of three-membered rings due to deviations from ideal bond angles.^3^ Consequently, “strain release” has been widely employed in organic synthesis as a powerful tactic to increase reaction rates, finding applications in total synthesis,^4^ polymer science,^5,6^ bioconjugation,^7,8^ and bioisosterism;^9,10^ it is also an important concept in biosynthesis (Figure 1a).^11^ However, despite the common belief that such pent-up strain energy fully explains the reactivity of species such as small rings, cycloalkynes, and cyclo-(*E*)-alkenes, even the simplest of these systems presents a paradox: cyclopropanes display markedly heightened ring-opening reactivity compared to cyclobutanes (*k*_rel_(cyclopropane) = 10^4^–10^7^ for intramolecular ring-opening reactions),^12^ despite having nearly identical strain energies (27.5 and 26.5 kcal mol^–1^, respectively).^3^

This puzzle has been the subject of extensive theoretical investigations. Stirling and co-workers^13^ proposed that cyclopropane relieves a larger proportion of angle strain (∼75%) than cyclobutane (∼50%) upon ring opening, while the groups of Hoz^14^ and Houk^15^ argued that differences in electronic structure (i.e., bonding) are instead the cause of the reactivity difference. Hoz proposed that rehybridization induced by bond angle compression enhances the electrophilicity of cyclopropane C–C bonds by lowering the energy of the σ* orbitals. On the other hand, Houk invoked an “orbital interactions through-bonds” (OITB)^20^ argument in which transition state (TS) aromaticity stabilizes ring-opening reactions of cyclopropane, whereas equivalent reactions of cyclobutane are destabilized due to an antiaromatic TS. While these explanations qualitatively explain the reactivity differences in these systems, a comprehensive predictive model connecting bonding to reactivity is yet to emerge.

We thus questioned whether the TS electronic structure and distinct reactivity of cyclopropane and other strained systems could be understood through commonly used models describing their ground state bonding.^21^ The Coulson–Moffitt “bent bonds” description,^22^ Walsh’s (p + sp^2^) rehybridization model,^23,24^ Dewar’s σ-aromaticity proposal,^25^ and Weinhold and Landis’ geminal hyperconjugation model^26^ all suggest that the valence electrons of cyclopropane are not confined to individual C–C σ bonds. Instead, they delocalize similarly to those in an aromatic π-system. This delocalization is illustrated by the higher dipole moment of chlorocyclobutane (2.20 D) compared with chlorocyclopropane (1.76 D), the latter being similar to that of chlorobenzene (1.60 D).^27,28^ While the importance of delocalization on the thermodynamic stability of systems containing conjugated π bonds, including aromatic rings, is universally accepted, its impact on bonding and reactivity in σ-frameworks, particularly in systems like cyclopropane, remains to be established.

In this work, we present a quantitative model to understand the interplay between delocalization, strain energy, and reactivity (Figure 1c). We propose that enhanced electronic delocalization within three-membered rings results in earlier, lower energy TSs, an effect that is distinct from barrier lowering due to strain release alone. This model not only accounts for the relative reactivity of cyclopropane and cyclobutane but also extends to *all* molecules containing one or more three-membered rings, including heterocycles and polycyclic structures. We demonstrate that in many cases, delocalization primarily governs reactivity, as seen in ring-opening reactions of bicyclo[1.1.0]butanes, [1.1.1]propellane, and epoxides.^29,30^ We establish a simple “rule of thumb” where each three-membered ring fused to the breaking bond lowers the activation barrier by ∼10 kcal mol^–1^, corresponding to a roughly 10^7^-fold rate enhancement at 298 K. This model also applies to “strain-promoted” azide-cycloalkyne (3 + 2) cycloadditions, commonly used as a bioconjugation strategy.^7^ Collectively, this framework unites the influence of strain-driven and delocalization-enabled reactivity and offers quantitative predictions of reaction barriers.

## Results and Discussion

### Model Construction

**Figure 2:**
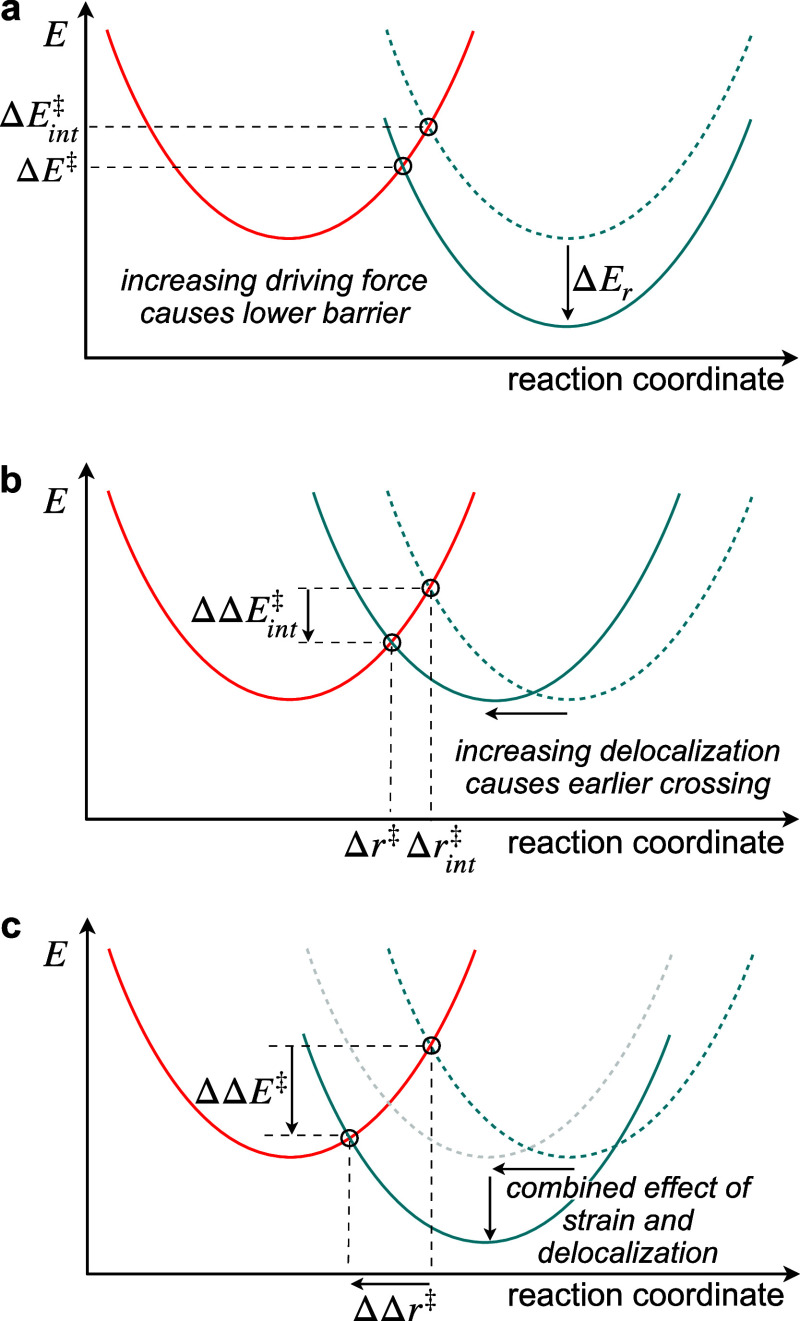
LFERs connecting strain release and reactivity. (a) According to Marcus theory, an increase in reaction driving force (Δ*E*_r_) causes an earlier curve crossing, lowering the TS energy (Δ*E*^‡^) relative to the intrinsic activation barrier (Δ*E*^‡^_int_). (b) Increasing bond delocalization decreases Δ*E*^‡^_int_, causing a lower energy, earlier curve crossing. (c) Increasing the reaction driving force and bond delocalization combine to enhance reactivity.

Our investigations began by establishing a linear free energy relationship (LFER) that connects strain release to reactivity. This LFER, a variant of the Marcus model (eq 1),^31,32^ represents breaking and forming bonds as intersecting parabolas defining the position of the TS on the reaction coordinate. For simplicity, following the original work by Marcus,^32^ the curvature of the breaking bond parabola is assumed to remain constant, which was found to be a reasonable first approximation for the reactions studied here (vide infra).^33^
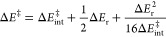
1Here, Δ*E*_r_ is the reaction driving force and Δ*E*^‡^_int_ denotes the intrinsic activation barrier when Δ*E*_r_ = 0. According to Hammond’s postulate, as Δ*E*_r_ becomes more negative, an earlier TS and a lower energy barrier are expected, depicted by the vertical movement of the product parabola relative to the reactant (Figure 2a).^34^ Truncating eq 1 at first order and introducing a proportionality constant, α, recovers the Bell–Evans–Polanyi (BEP) principle (eq 2),^35,36^ where the activation barrier (Δ*E*^‡^) is assumed to vary linearly with the reaction driving force (Δ*E*_*r*_) between two reactions.
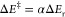
2

For similar reactions with equal driving forces, the difference in Δ*E*^‡^ is simply the difference in Δ*E*^‡^_int_ (i.e., ΔΔ*E*^‡^ = ΔΔ*E*^‡^_int_), represented by the horizontal displacement of the product parabola. Consequently, an earlier TS implies a lower activation energy (Figure 2b). In the context of small-ring reactivity, we propose that electron delocalization within three-membered rings reduces this intrinsic activation barrier by increasing the polarizability of the ground state electron density, compared with four-membered ring analogues. Hait and Head-Gordon recently demonstrated that the polarizability of the electron density is maximized at or near a TS due to electron delocalization accompanying partial bond cleavage and formation.^37^ Therefore, the relationship between delocalization and reactivity can be qualitatively understood by considering how delocalization evolves during a bond breaking/making process: reaching a delocalized electron arrangement at the TS is facilitated if the relevant bond(s) are already partially delocalized in the ground state, resulting in an earlier, lower-energy TS.

We may combine eq 1 with the relationship Δ*E*^‡^_int_ = Δ*E*^‡^_int_(0) + ΔΔ*E*^‡^_int_, where Δ*E*^‡^_int_(0) is the reference intrinsic activation barrier in the absence of a driving force or delocalization contribution, to capture both strain release and delocalization effects within the Marcus formalism. A further substitution of ΔΔ*E*^‡^_int_ = βχ is made, where χ represents bond delocalization and β is a proportionality constant, to enable simple calculation of the contribution of delocalization using electronic structure calculations (vide infra). The resultant equation (eq 3) accounts for the contribution of both the reaction driving force (through Δ*E*_r_) and the intrinsic activation barrier (through χ) to the activation energy (Figure 2c, see the SI for full derivation).^38^ Values for α and β are empirical parameters that can be determined using multiple linear regression (MLR) and can be thought of as sensitivity constants for a given reaction type. These parameters will reveal the relative importance of strain release and delocalization in a given reaction type and can also be compared between reaction types to uncover fundamental differences between reactivity modes.
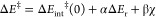
3

To quantify the extent of electron delocalization and its effect on reactivity, we employed both an orbital-based and a density-based approach. First, we calculated the occupation number (*N*_occ_) of the natural bond orbital (NBO) corresponding to the breaking bond. Deviation from a full occupation of 2 (denoted as 2–*N*_occ_) describes the extent of ground-state bond delocalization (i.e., χ_NBO_ = 2–*N*_occ_).^39^ For example, in cyclopropane, electron donation from a breaking C–C σ into a geminal σ* orbital increases χ_NBO_, capturing the hyperconjugation (delocalization) effect proposed by Weinhold and Landis (Figure 1b). Additionally, we computed the ratio χ_ρ_ = *D*_σ_/*D*_σ_^0^ [used to calculate the electron localization function, ELF = (1 + χ_ρ_^2^)^−1^], which measures the excess kinetic energy density due to Pauli repulsions (*D*_σ_) relative to the uniform electron gas, *D*_σ_^0^.^40^ As for the χ_NBO_ parameter, increasing values of χ_ρ_ indicate increasingly delocalized electrons. As a result, we expect the sensitivity constant β to be negative if increasing delocalization causes a lower activation barrier. The close agreement between the reactivity models derived from the conceptually distinct χ_NBO_ and χ_ρ_ parameters suggests that the effect of delocalization on reactivity is correctly captured (vide infra). In summary, we anticipate a decrease in Δ*E*^‡^ either through an increase in driving force (α > 0, as predicted by the BEP principle) and/or an increase in bond delocalization (β < 0).

### Polycyclic Hydrocarbon Ring Opening

**Figure 3:**
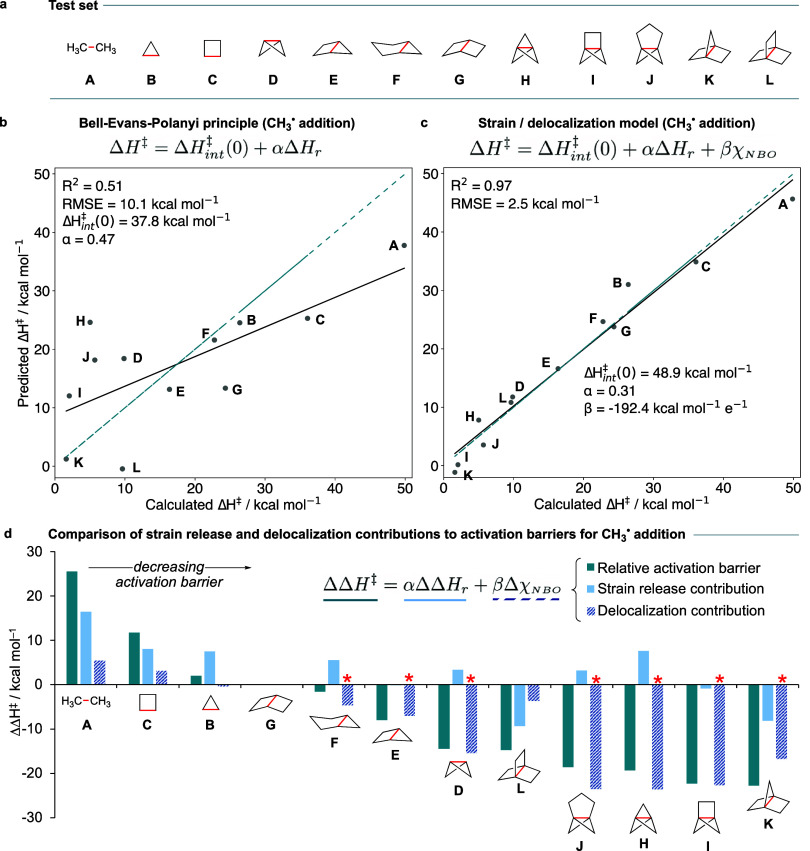
Delocalization dominates trends in “strain release” ring-opening reactions. (a) Test set of acyclic, monocyclic, and fused polycyclic hydrocarbons. (b) BEP plot (predicted vs calculated Δ*H*^‡^, kcal mol^–1^) for the addition of methyl radical to the red bonds of the molecules in the test set. The blue dashed line denotes perfect correlation. (c) Prediction of Δ*H*^‡^ from Δ*H*_r_ and χ_NBO_ (eq 3). (d) Breakdown of strain and delocalization (χ_NBO_) contributions to ΔΔ*H*^‡^ (kcal mol^–1^) for the addition of methyl radical to the test set, relative to bicyclo[2.2.0]hexane (**G**), with α = 0.51 and β **= −**192.4 kcal mol^–1^ e^–1^. Asterisks indicate the cases where delocalization dominates over strain release.

To explore the importance of delocalization on the reactivity of small rings, activation and reaction enthalpies (Δ*H*^‡^ and Δ*H*_r_) were calculated for the addition of methyl radical to a test set of 12 acyclic, monocyclic, and fused polycyclic hydrocarbons with ring sizes varying from three to five (Figure 3a). Predicted Δ*H*^‡^ values, obtained via either the BEP principle (eq 2) or our strain/delocalization model (eq 3), were compared to QM-computed enthalpies (calculated Δ*H*^‡^). Entropic effects on reactivity differences are negligible for these reactions, illustrated by the close agreement between relative activation enthalpies and Gibbs free energies (see the Supporting Information).

Applying the BEP principle (eq 2) to this set revealed that, as anticipated, Δ*H*_r_ alone inadequately predicts reactivity (Figure 3b), with a poor correlation (*R*^2^ = 0.51) and a root-mean-squared error (RMSE) of 10.1 kcal mol^–1^. Notably, [1.1.1]propellane (**H**), cyclopropane (**B**), and cyclobutane (**C**) (Δ*H*^‡^ = 5.0, 26.4, and 36.1 kcal mol^–1^) exhibit a significant span of activation enthalpies (>30 kcal mol^–1^) despite similar reaction enthalpies (Δ*H*_r_ = −28.2, –28.4, and −26.8 kcal mol^–1^).^41^ However, linear relationships do appear when considering reactions in which the number of cyclopropane rings is equal, for example **A**/**C**/**G**/**L** (0 cyclopropane rings) vs **B**, **F**, **E**, **K** (1 cyclopropane ring).

In line with the anticipated relationship between delocalization and reactivity introduced above, integrating bond delocalization (χ_NBO_ = 2–*N*_occ_) using eq 3 resulted in an excellent correlation between predicted and calculated activation enthalpies (Figure 3c, *R*^2^ = 0.97) and low RMSE (2.5 kcal mol^–1^). The negative value of the “delocalization coefficient” β (−192 kcal mol^–1^ e^–1^) reflects the decrease in the intrinsic barrier with increasing delocalization.

Inclusion of the χ_NBO_ parameter alongside Δ*H*_r_^2^ leads to near-identical results (Figure S2). Employing the density-based delocalization parameter χ_ρ_ was similarly successful in predicting activation barriers (*R*^2^ = 0.94, RMSE = 3.3 kcal mol^–1^, Figure S3), supporting the interpretation that the localized NBO descriptor effectively captures the electron delocalization effect. Notably, descriptors based on canonical orbital properties (e.g., HOMO–LUMO gap) gave unphysical results (Figures S4–S6), such as negative intrinsic activation barriers. These results not only confirm that our model improves the originally poor correlation obtained by the BEP principle but also provides a physically grounded explanation of the connection between χ and electron delocalization, as illustrated by these orbital and density analyses.

To directly compare the impact of delocalization on activation barriers, we examined changes in barrier (ΔΔ*H*^‡^ = αΔΔ*H*_r_ + βΔχ_NBO_) for the test set relative to bicyclo[2.2.0]hexane (**G**, Figure 3d), which exhibits a moderate strain release value (−52.5 kcal mol^–1^) but has a small χ_NBO_ value (0.045 *e*). Among the test set, *delocalization* (quantified by the χ_NBO_ term, eq 3), *not strain release, emerged as the primary cause of reactivity difference for seven of the 12 members relative to***G** (denoted by asterisks). In four cases (**D**, **F**, **H**, and **J**), the overall favorable ΔΔ*H*^‡^ arises from a large delocalization contribution, which overcomes the unfavorable change in strain energy relative to **G**. It is especially notable that for the classic “strain release” reagents bicyclo[1.1.0]butane (**D**) and [1.1.1]propellane (**H**), ring strain *increases* the reaction barriers by 3.4 and 7.6 kcal mol^–1^, respectively; the barrier-lowering delocalization effects of −15.4 and −23.5 kcal mol^–1^ are therefore not only essential but also the fundamental basis of their “spring-loaded” behavior. We note that ΔΔ*H*^‡^ is not exactly equal to the sum of strain release and delocalization effects due to other small contributions not included in this model (vide infra).

**Figure 4:**
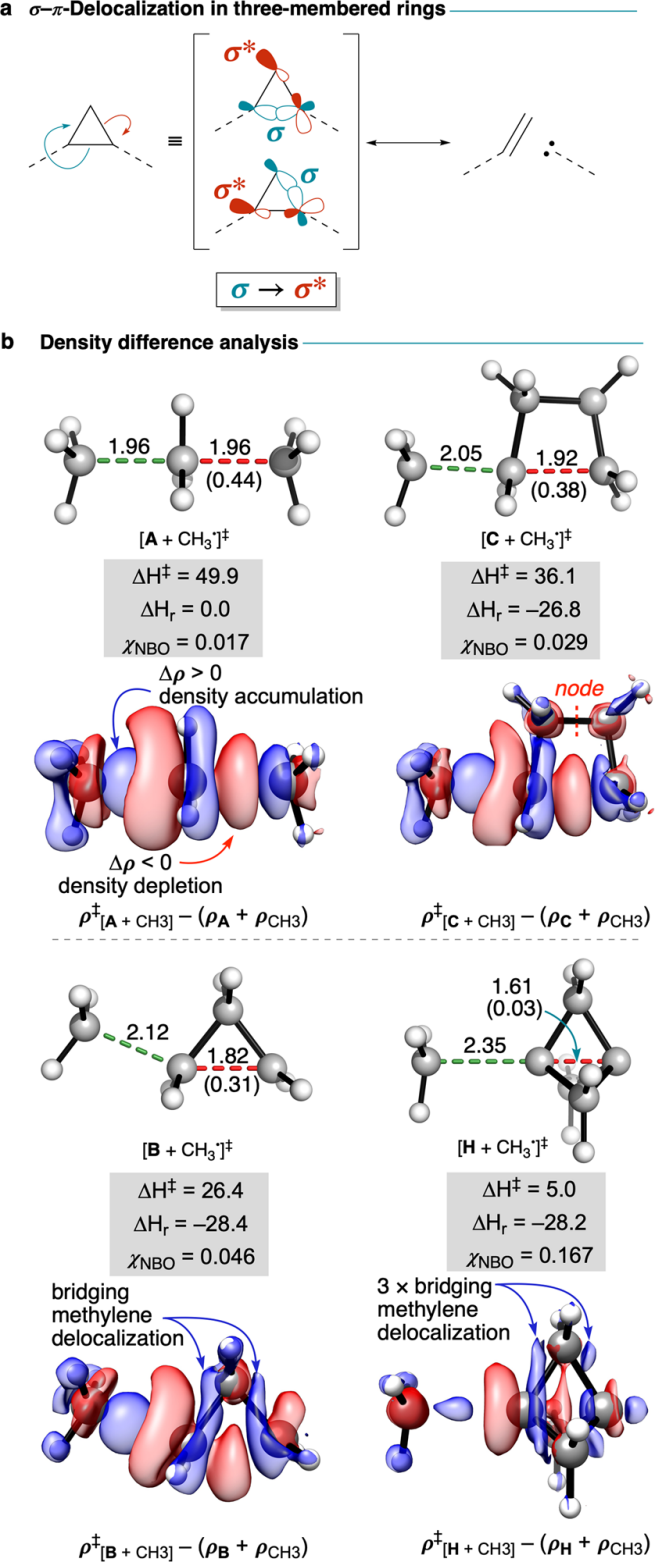
(a) Selected TS geometries (distances in Å), enthalpies (kcal mol^–1^), χ_NBO_ values (*e*), and EDD plots (isovalue of 0.015 *e* Å^–3^) for the addition of methyl radical to ethane, cyclobutane, cyclopropane, and [1.1.1]propellane. Difference between TS and equilibrium bond lengths (Δ*r*^‡^) is shown in parentheses. (b) General σ–π-delocalization model proposed for three-membered rings.

The origins of delocalization-enabled reactivity in small rings may be understood using the concept of σ–π-delocalization (Figure 4a).^29^ Electrons are delocalized over methylene groups via geminal σ → σ* hyperconjugation (i.e., through-bond communication) that is facilitated by the p orbital overlap. This hyperconjugation is substantial in three-membered rings and increases as the σ bond becomes more “inverted”. However, delocalization is negligible in four-membered rings due to geometric and symmetry constraints. The presence or absence of σ–π-delocalization—and therefore the importance of delocalization to lower activation barriers—can be predicted simply by counting the number of cyclopropane rings fused to the breaking bond.

The relationship between this σ–π-delocalization effect and the reactivity of small rings can be visualized by plotting the electron density difference (EDD) between the total TS electron density and the densities of each distorted fragment at the TS, for a series of C–C bond cleavage reactions (Figure 4b). For the reaction of methyl radical with ethane, the EDD plot involves the expected removal of electron density from the breaking C–C bond (red lobes) and accumulation in the forming C–C bond (blue lobes). Similar behavior is observed with cyclobutane, with a node between the bridging methylenes indicating a lack of through-bond communication. However, for cyclopropane, a buildup of electron density on the bridging methylene indicates stabilizing delocalization. [1.1.1]Propellane shows an equivalent effect, where delocalization now extends across all three bridging methylene groups and the bridgehead carbon atoms.

It is interesting to note that while the interbridgehead bond in [1.1.1]propellane can be described as a charge-shift bond,^42^ the origins of its *reactivity* are thus no different to those of the covalent bonds of, for instance, cyclopropane; it is simply the combination of the strain release driving force and the ability to delocalize electrons over an additional two methylene groups that explains the reactivity differences.

### Structure–Reactivity Relationship

Informed by the σ–π-delocalization model, we next investigated whether the *number* of three-membered rings fused to the breaking bond alone (*n*_3_) could serve as a metric for delocalization (eq 4).
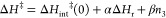
4

**Figure 5:**
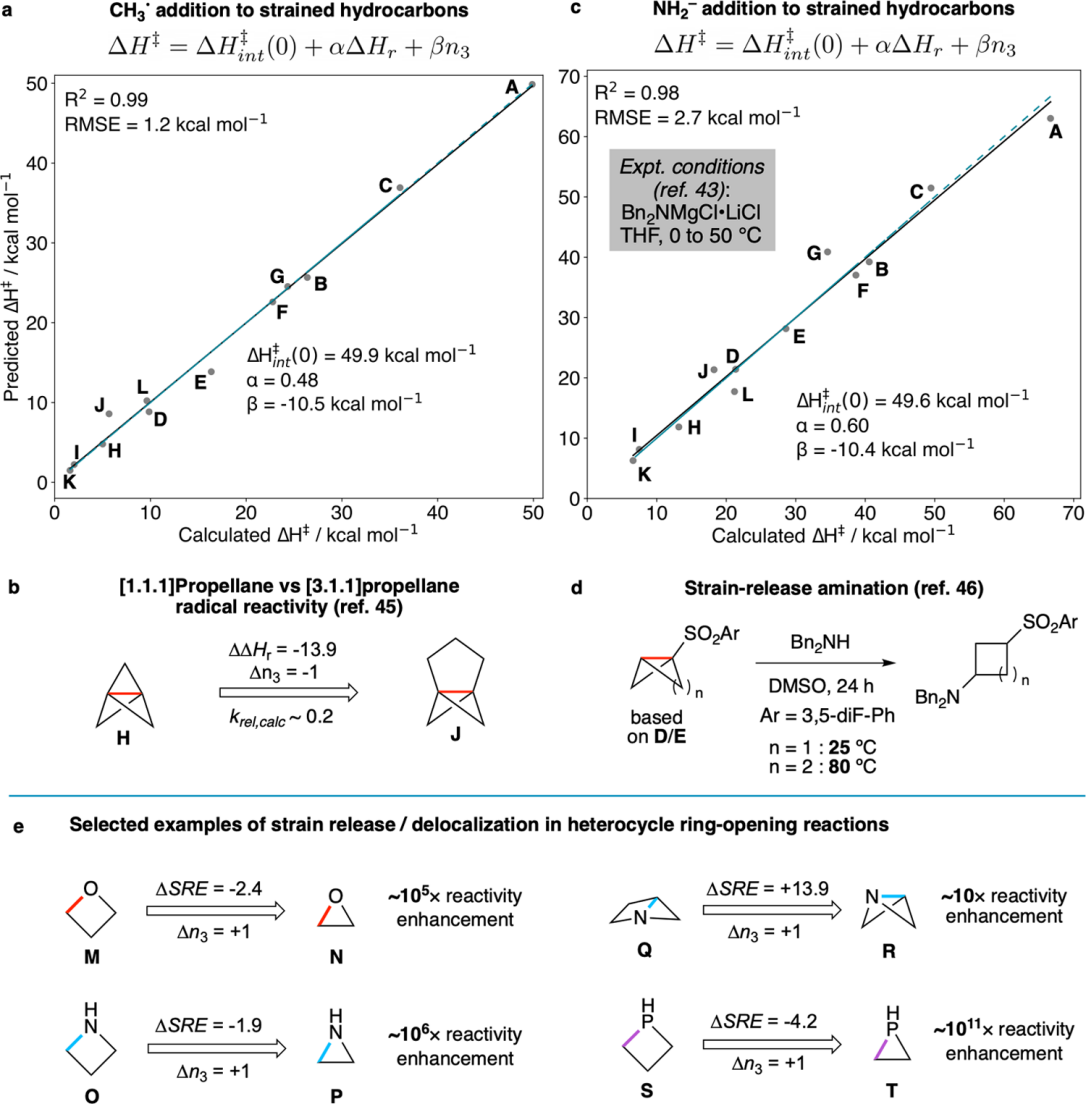
Implications of strain and delocalization on general reactivity. MLR plots for the prediction of Δ*H*^‡^ from Δ*H*_r_ and *n*_3_ for the hydrocarbon test set with CH_3_^•^ (a) and NH_2_^–^ (b) using eq 4. The blue dashed lines denote perfect correlation. (c) Increasing strain release driving force for [3.1.1]propellane (**J**) vs [1.1.1]propellane (**H**) counteracts the decrease in intrinsic reactivity due to a loss of bond delocalization, resulting in similar reactivity. (d) Addition of dibenzylamine to bicyclo[1.1.0]butane and bicyclo[2.1.0]pentane sulfones. Increased delocalisation lowers the required temperature for this reaction, opposing the expected behavior based on strain release energies alone. . (e) Selected examples of the synergy or antagonism between strain release and delocalization in the ring-opening reactivity of heterocycles. See the SI (Figures S9 and S10) for Marcus *E*_a_ values from refs (47) and (48) and the full data set of radical and anionic reactivity. All relative reaction rates were estimated at 298 K.

Substituting χ = *n*_3_ in eq 3 accurately predicts reactivity (Figure 5a). Specifically, each three-membered ring fused to the breaking C–C bond reduces the intrinsic activation energy by ∼10 kcal mol^–1^, corresponding to a ∼10^7^-fold increase in the rate constant at 298 K. This simple model effectively captures the greater reactivity of cyclopropane over cyclobutane and also the contrasting reactivities of [1.1.1]propellane and cyclopropane; the increased reactivity of the former is attributed to a greater number of three-membered rings fused to the breaking bond (*n*_3_ = 3). Varying the number of three-membered rings fused to a breaking bond therefore offers a simple way to modulate the reactivity of the system—for example, switching the behavior of a molecule from a highly reactive bioconjugation warhead (e.g., bicyclo[1.1.0]butanes similar to **D**)^8,9,43^ to an inert lipid tail group (e.g., bicyclo[2.2.0]hexane “ladderanes” based on **G**).^44^

We recently applied this concept to develop the radical ring-opening reactivity of [3.1.1]propellane (**J**).^45^ Compared with [1.1.1]propellane, [3.1.1]propellane sacrifices bond delocalization (*n*_3_ = 3 vs 2, respectively) for an increased driving force (Δ*H*_r_ = −28.2 vs −42.1 kcal mol^–1^, respectively, Figure 5b). The predicted difference in Δ*H*^‡^ between these systems for a radical addition is only 3.8 kcal mol^–1^, in reasonable agreement with the calculated value of 1.1 kcal mol^–1^ (*k*_rel,calc_ ∼ 0.2 at 298 K).^45^ This result suggests that decreasing delocalization but increasing strain release coincidentally results in similar radical reactivity to [1.1.1]propellane. Pleasingly, [3.1.1]propellane was found to be a viable substrate for numerous radical reactions previously developed for [1.1.1]propellane, including atom transfer radical additions, dual photoredox/Cu catalysis, and chalcogen atom addition reactions.^45^

The delocalization model (eq 4) can also be applied to two-electron processes, such as the nucleophilic addition of amide anions to **D**, **E**, and **H**.^43,46^ When using NH_2_^–^ as a model nucleophile, an excellent correlation and low error were observed between predicted and calculated activation enthalpies (*R*^2^ = 0.98, RMSE = 2.7 kcal mol^–1^, Figure 5c). The β coefficient (−10.4 kcal mol^–1^) is almost identical to the one-electron reaction, supporting the idea that delocalization-modulated reactivity is intrinsic to the bonding pattern found in the small rings. If delocalization effects were absent, the barrier to nucleophilic addition to [1.1.1]propellane would increase by ∼30 kcal mol^–1^, rendering it inert under the reaction conditions.

In other words, strain release alone cannot account for the observed reactivity—delocalization again emerges as the primary driver of reactivity. This principle holds true for bicyclo[1.1.0]butanes (**D**) and bicyclo[2.1.0]pentanes (housanes, **E**), where activation barriers would increase by ∼20 and ∼10 kcal mol^–1^, respectively, in the absence of delocalization. This effect is corroborated by experimental results on the addition of dibenzylamine across the interbridgehead bonds of bicyclo[1.1.0]butane and bicyclo[2.1.0]pentane sulfones (Figure 5d), where the former affords the cyclobutylamine product at ambient temperature, whereas the latter requires heating to 80 °C to form the equivalent cyclopentane.^46^ This reactivity difference directly opposes the behavior expected solely based on strain release energies (i.e., thermodynamics) alone (−40.2 and −48.1 kcal mol^–1^ for bicyclo[1.1.0]butane and bicyclo[2.1.0]pentane, respectively).

### Heterocycle Ring Opening

We next extended the model in eq 4 to radical and anionic ring-opening reactions of heterocyclic systems with various bond types (C–C, C–N, C–O, C–P, and C–S), previously studied by Hoz and co-workers.^47,48^ Notably, three-membered rings consistently exhibit higher reactivity than four-membered homologues due to pronounced bond delocalization. For instance, the anionic ring-opening rate for ethylene oxide **N** (*n*_3_ = 1) is ∼10^5^ times greater than that of oxetane **M** (*n*_3_ = 0), despite only a 2.4 kcal mol^–1^ difference in strain release energies (Figure 5e). This reactivity difference underscores the utility of epoxides in synthesis and biosynthesis^5,11^ and may explain the success of oxetanes as biostable motifs in drug discovery.^30^ Similarly, aziridine **P** undergoes nucleophilic ring opening ∼10^6^ times faster than azetidine **O**, primarily due to delocalization effects in the breaking of its three-membered ring. Remarkably, despite azabicyclo[2.1.0]pentane **Q** (*n*_3_ = 1) releasing almost 14 kcal mol^–1^ more strain energy than azabicyclo[1.1.0]butane **R** (*n*_3_ = 2) upon nucleophilic ring opening, the latter molecule is predicted to be similarly reactive due to increased delocalization.

Interestingly, heterocycles containing third-row heteroatoms (e.g., phosphorus and sulfur) are more sensitive to the number of three-membered rings than their second-row counterparts, resulting in far greater predicted ring-opening reactivity of (unknown) epiphosphine **T** than phosphetane **S** (Figure 5e). This sensitivity increase can be attributed to the higher polarizability of third-row atoms,^49^ facilitating additional electron delocalization at the TS compared to second-row elements.^37^

### Rule of Thumb for Reactivity Prediction

A “rule of thumb” for rapidly estimating relative reactivity between two substrates (ΔΔ*H*^‡^) can be derived using the difference in strain release energies (ΔSRE) between a pair of substrates (tabulated in https://github.com/duartegroup/strain-delocalisation and Figure S11), the difference in the number of three-membered rings fused to the breaking bonds for this pair of substrates (Δ*n*_3_), and considering α = 0.5 and β = −10 kcal mol^–1^ (based on the results for radicals and anions obtained above).
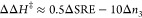
5

**Figure 6:**
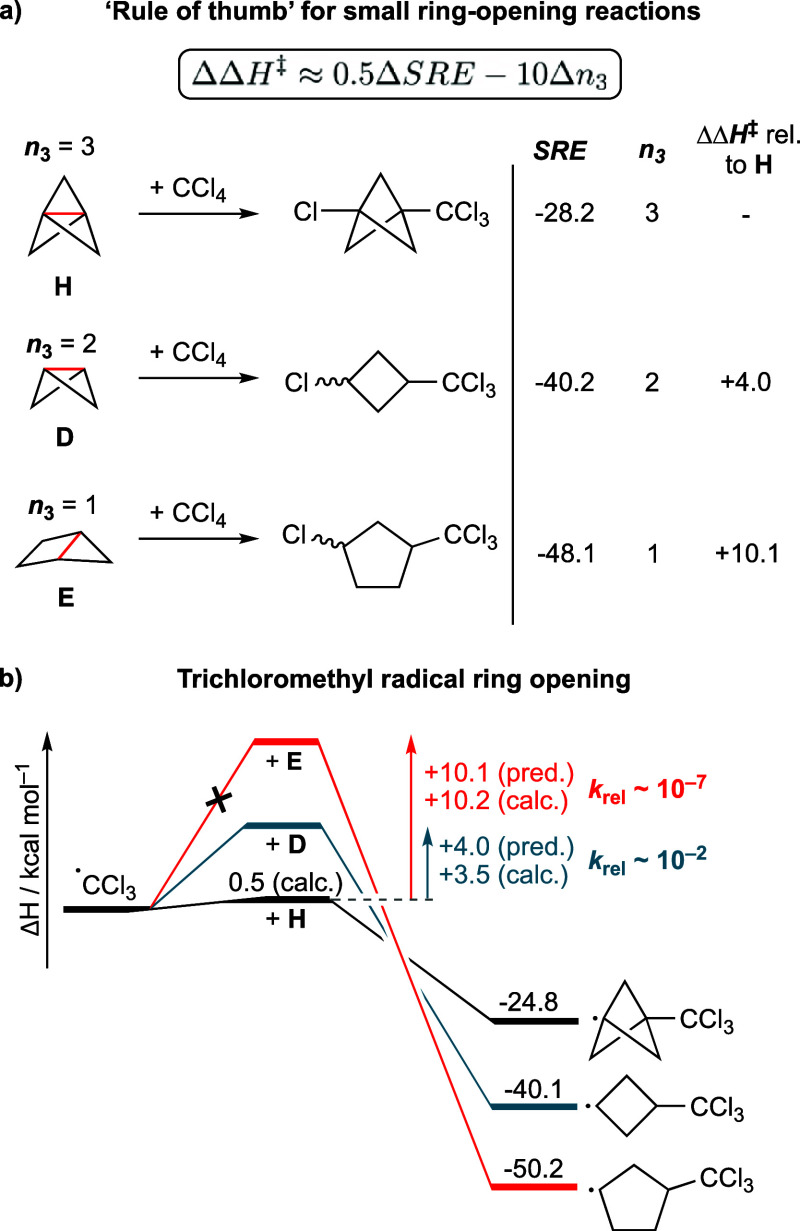
Applications of the rule of thumb. (a) Predicted relative activation enthalpies (ΔΔ*H*^‡^, kcal mol^–1^) based on SRE and *n*_3_ using eq 5. (b) Comparison of estimated and calculated ring-opening activation enthalpies (Δ*H*, kcal mol^–1^) using eq 5.

This model is easily applicable to rationalize differences in reactivity for the radical addition reactions of [1.1.1]propellane (**H**), bicyclo[1.1.0]butane (**D**), and bicyclo[2.1.0]pentane (**E**) with BrCCl_3_ or CCl_4_ (Figure 6). While **H** and **D** readily undergo addition of the trichloromethyl radical, **E** does not.^50^ Additional competition reactions demonstrate that **H** undergoes significantly more rapid reaction than **D**. SREs alone fail to explain this reactivity pattern, but our rule of thumb (eq 5) correctly predicts the observed trend (Figure 6a). The estimated activation enthalpies for **D** and **E** are 4.0 and 10.1 kcal mol^–1^ higher than **H**, respectively, in line with calculated values of 3.5 and 10.2 kcal mol^–1^ (Figure 6b). These barriers translate to relative addition rates (*k*_rel_) that are ∼10^2^ and ∼10^7^ times slower for **D** and **E** than **H** at 298 K—entirely suppressing reactivity in the case of **E**.

Estimating the relative reactivity of bicyclo[1.1.0]butane and bicyclo[2.1.0]pentane sulfones offers a further example of application of the model (Figure 5c); the model suggests that the greater strain released in the ring opening of the bicyclo[2.1.0]pentane should be offset by the greater delocalization in the (more reactive) bicyclo[1.1.0]butane. The strain release contribution to the TS barrier change, 0.5ΔSRE, is approximately +4 kcal mol^–1^ (half the difference between −40.2 and −48.1), and the delocalization contribution, Δ*n*_3_, is approximately −10 kcal mol^–1^ (from the difference of one three-membered ring), leading to a 6 kcal mol^–1^ lower TS barrier for bicyclo[1.1.0]butane than bicyclo[2.1.0]pentane. From the reported reaction conditions,^46^ ΔΔ*G*^‡^ can be roughly estimated as 5 kcal mol^–1^ (see Section S6 for further discussion), which is only a 1 kcal mol^–1^ difference from the rule of thumb prediction. In short, the enhanced reactivity of bicyclo[1.1.0]butane compared with bicyclo[2.1.0]pentane can therefore be predicted simply by looking up SRE values and counting the number of three-membered rings.

### Extension to Cycloaddition Reactions

**Figure 7:**
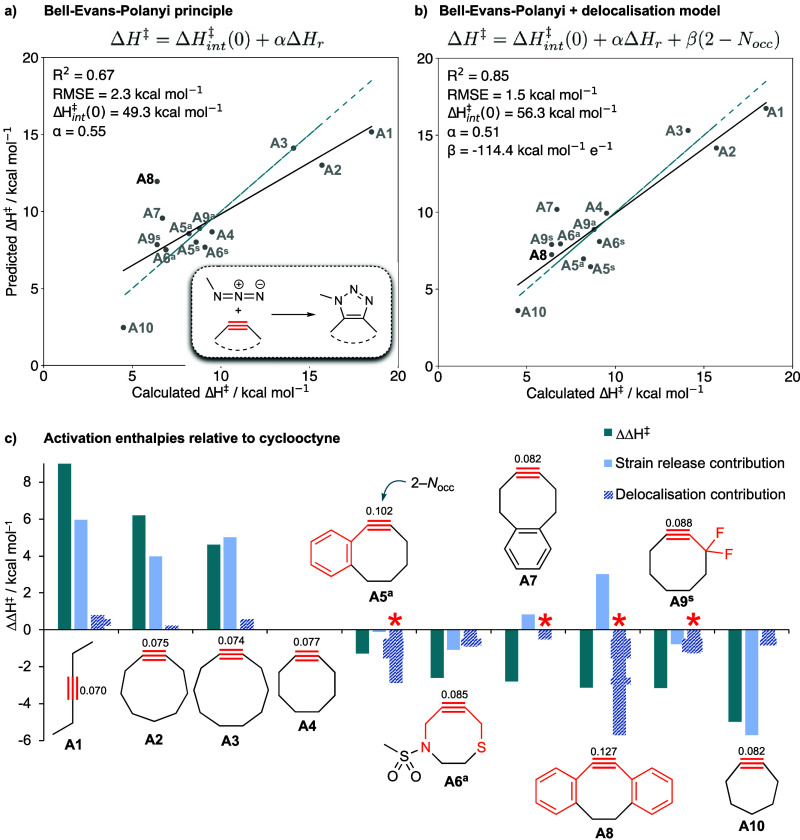
Application to (3 + 2) azide–alkyne cycloaddition reactions. Delocalization, not strain release, explains the enhanced reactivity of dibenzocyclooctyne over cyclooctyne in (3 + 2) cycloadditions with methyl azide. (a) BEP plot (predicted vs calculated Δ*H*^‡^, kcal mol^–1^) for the addition of methyl azide to the red bonds of the alkynes in the test set. The blue dashed line denotes perfect correlation. (b) Prediction of Δ*H*^‡^ from Δ*H*_r_ and χ_NBO_ (eq 3). (c) Breakdown of strain release and delocalization (χ_NBO_) contributions to ΔΔ*H*^‡^ (kcal mol^–1^) for the addition of methyl azide to the test set relative to cyclooctyne (**A4**), following the protocol in Figure 3d. Asterisks indicate the cases where delocalization dominates over strain release, and superscripts ^a^ and ^s^ refer to *anti* and *syn* TSs, respectively.

This model can be extended beyond three-membered ring cleavage where TS electronic delocalization can operate simultaneously with strain release. We compiled a data set encompassing strain release energies and χ_NBO_ (= 2–*N*_occ_) values for various bond types across commonly employed strained molecules, including carbocycles, heterocycles, cycloalkynes, and cycloalkenes (Figure S11 and https://github.com/duartegroup/strain-delocalisation). For example, the principle that more delocalized bonds are inherently more reactive applies to reactions such as strain-releasing “click” (3 + 2) azide–alkyne cycloadditions.^51^ Strategies to accelerate such reactions primarily focus on increasing the strain of the alkyne, such as by incorporating the alkyne into a medium-sized ring.^52,53^ Significant efforts have been undertaken to understand reactivity patterns using the distortion/interaction-activation/strain (DI-AS) model, which has identified alkyne distortion and greater interfragment interactions as factors that reduce TS barriers.^54,55^ A BEP analysis of the cycloaddition between methyl azide and a range of alkynes (Figure 7a) reveals a loose correlation (*R*^2^ = 0.67) between the reaction driving force (Δ*H*_r_) and the activation barrier (Δ*H*^‡^) with a reasonably low RMSE (2.3 kcal mol^–1^). This result suggests that, in general, strain release enhances alkyne reactivity, causing faster cycloadditions as the ring size decreases from 10 (**A3**) to 7 (**A10**)^56,57^.

However, as noted by Harris and Alabugin,^52^ an exception to this relationship is dibenzocyclooctyne **A8.** This compound was designed to enhance strain, and consequently reactivity, by increasing the number of sp^2^ centers in the medium ring. In fact, **A8** is more reactive than its strain release energy alone suggests. The reaction enthalpy for **A8** is 6 kcal mol^–1^*less exothermic* than the parent cyclooctyne **A4**, which should in principle *increase* its activation barrier relative to **A4** by around 3 kcal mol^–1^ if strain release alone were to govern reactivity. Dissecting ΔΔ*H*^‡^ between **A8** and **A4** into strain release and delocalization components (using the same approach as shown in Figure 3d) reveals that enhanced delocalization due to greater π-conjugation (Δχ_NBO_ = 0.05 *e*) in **A8** accounts for a 6 kcal mol^–1^ barrier-lowering effect. Consequently, *delocalization counteracts the effect of decreased strain release observed in***A8**, resulting in a net lowering of the activation barrier by 3 kcal mol^–1^—an approximate 10^3^-fold rate acceleration at 298 K compared with **A4**. A similar analysis across a set of cycloalkynes (Figure 7b,c) reveals the importance of delocalization on the reactivity of monobenzocyclooctynes (**A5**), and to a smaller extent difluorinated cyclooctyne **A9** and distal benzocyclooctyne **A7**, denoted by red asterisks in Figure 7c. As with the small ring-opening reactions discussed above (Figure 3c), the negative sign of the delocalization coefficient β for this cycloaddition reaction (−114 kcal mol^–1^ e^–1^) reflects the decrease in the intrinsic barrier due to delocalization. The smaller magnitude of β for the cycloaddition reaction compared with the small-ring opening (−114 vs −192 kcal mol^–1^ e^–1^, respectively, for χ_NBO_) reflects the lower sensitivity of the cycloaddition toward variation in bond delocalization. We suggest that this lower sensitivity may arise from a smaller orbital overlap between the breaking π bond and the hyperconjugating group such that the effect of this electron delocalization on the TS is less pronounced.

Like any empirical model, there are limitations to the accuracy achievable with this model, since other factors, such as dipole effects and noncovalent interactions present at the TS but not in the reactant state, and explicit variation in bond force constants, are neglected. Incorporating these factors could improve accuracy through the inclusion of further descriptors. However, the overall improvement in barrier height prediction (*R*^2^ = 0.85, RMSE = 1.5 kcal mol^–1^, Figure 7b) compared with the BEP model (*R*^2^ = 0.67, RMSE = 2.3 kcal mol^–1^, Figure 7a) illustrates the generality and importance of delocalization on reactivity across a range of organic reactions using only a small number of physical effects. Comparing results of conventional DI-AS analysis to our delocalization model shows that more delocalized breaking bonds require less distortion to adopt the TS geometry, leading to an earlier TS. Likewise, greater delocalization could facilitate stronger electronic interactions between reactants due to an enhanced orbital overlap earlier along the reaction coordinate. A drawback of the DI-AS approach is the necessity for explicit knowledge of the TS geometry and energy, whereas our model enables a quick and quantitative estimation of reactivity using solely ground state properties. This feature is anticipated to be valuable when designing new “strain-release”-driven reactions.

## Conclusions

Strain energy is often invoked to rationalize observed reactivity patterns and is commonly cited as the cause of the heightened reactivity of small carbo- and heterocyclic rings and cycloalkynes. Through analysis of radical and nucleophilic additions to small rings and azide/cycloalkyne click reactions, strain release is shown to be important but insufficient factor to promote these facile reactions. Here, we have introduced the concept of “bond delocalization”, manifested through electronic effects such as (hyper)conjugation, to enrich our understanding of the complex relationship between structure, bonding, and reactivity in a diverse array of reactions. We suggest that more delocalized bonds are intrinsically more reactive, an effect completely independent of their strength. In several cases, this bond delocalization effect is shown to dominate the strain release effects that were previously assumed to be the origin of the “spring-loaded” reactivity, for example explaining the facile reactivity of bi- and tricyclic alkanes and conjugated cycloalkynes. To aid the integration of these ideas into novel “strain release” strategies, a simple model has been developed that offers rapid and quantitative reactivity predictions.

## Methods

Quantum chemical calculations were run using ORCA (v 4.2.1)^69^ at the [DLPNO–CCSD(T)/def2-QZVPP (TightPNO)//B2PLYP-D3BJ/def2-TZVP] level of theory (CH_3_^•^ reactions) or [SMD(THF)/DLPNO–CCSD(T)/ma-def2-QZVPP (TightPNO)//SMD(THF)/B2PLYP-D3BJ/def2-TZVP (ma-def2-TZVP on N)] level of theory (NH_2_^–^ reactions).^58,73−76^ Strain release energies were obtained at the [DLPNO–CCSD(T)/def2-QZVPP (TightPNO)//B2PLYP-D3BJ/def2-TZVP] level of theory.^59−62^ Alkyne (3 + 2) cycloadditions were calculated at the B2PLYP-D3BJ/def2-TZVP level. NBO occupation numbers were calculated using the NBO program (v 7.0) based on the relaxed density, and density-based descriptors were calculated with Multiwfn (v 3.6).^63^ All data processing was carried out using the *Scikit-learn* package with Python 3.7.^64^ Enthalpies were chosen for a direct comparison with strain energies, which are commonly reported instead of Gibbs free energies.^65−68^ Trends in enthalpy and Gibbs free energy were found to be in excellent agreement for all reactions studied here.^70−72^ For further details, see the Supplementary Methods.

## Data Availability

The data underlying this study are available free of charge on the ACS Publications Web site and also at https://github.com/duartegroup/strain-delocalisation.

## Supporting Information Available

Details of calculation methods, supplementary discussion (Sections S1–S7), MLR plots, calculated and predicted activation enthalpies, differences in thermodynamic quantities, anionic and radical reactions, set of strain release energies, balanced hydrogen transfer reactions, and delocalization values (PDF)
Readme file summarizing supporting data that accompanies the manuscript, xyz coordinates and energies of all stationary points, code to generate all linear regression data, and plots discussed in this paper (ZIP)
