# Supplementary material for: Beyond Strain Release: Delocalization-Enabled Organic Reactivity
Source: J Org Chem. 2024 Jul 6;89(14):9979–89. doi: 10.1021/acs.joc.4c00857 (PMC11267611; doi:10.1021/acs.joc.4c00857)
Supplement: Supplementary file 1 — jo4c00857_si_001.pdf [file jo4c00857_si_001.pdf]

Supporting information for "Beyond strain release: Delocalization-enabled organic reactivity"

Alistair J. Sterling,[a,b]* Russell C. Smith,[c] Edward A. Anderson[a]* & Fernanda Duarte[a]*

[a]Chemistry Research Laboratory, University of Oxford, 12 Mansfield Road, Oxford OX1 3TA, UK
[b]Department of Chemistry & Biochemistry, The University of Texas at Dallas, 800 W. Campbell Rd., Richardson, TX 75080, USA
[c]Abbvie Drug Discovery Science & Technology (DDST), 1 North Waukegan Road, North Chicago, IL 60064, USA

E-mail: fernanda.duartegonzalez@chem.ox.ac.uk; edward.anderson@chem.ox.ac.uk; alistair.sterling@utdallas.edu

Contents

S1 Computational methods **S2**

S2 Linear free energy and geometric relationships **S3**

S3 Hydrocarbon ring-opening reactivity **S6**

S4 Heterocycle ring-opening reactivity **S14**

S5 Tabulated strain release energies and delocalization values **S16**

S6 'Rule of thumb' worked example **S17**

S7 Azide-alkyne (3+2) cycloaddition reactivity **S27**

S8 References **S29**

S1 Computational methods

All energies and thermodynamic quantities reported in this work were obtained using ORCA (v. 4.2.1).[1] Minima and transition states (TSs) were initially identified using *autodE* (v. 1.0.0b3),[2] with low energy conformers located using the ETKDGv2 algorithm implemented in RDKit v. 2019.03.4,[3] and optimised using GFN2-xTB implemented in xTB (v 6.2.2)[4] followed by PBE0-D3BJ/def2-TZVP//PBE0-D3BJ/def2-SVP in ORCA (v. 4.2.1).[1,5,6] Anionic reactions were run using the GBSA[7] / CPCM[8] solvent models for THF in xTB / ORCA, respectively. Geometries and energy were then refined in ORCA at the [DLPNO-CCSD(T)/def2-QZVPP (TightPNO)//B2PLYP-D3BJ/def2-TZVP] level of theory ($CH_3^{\bullet}$ reactions) or [SMD(THF)-DLPNO-CCSD(T)/ma-def2-QZVPP (TightPNO)// SMD(THF)-B2PLYP-D3BJ/def2-TZVP (ma-def2-TZVP on N)] level of theory (NH_2^- reactions).[5,6,9,10] All calculations used the resolution of the identity approximation (RIJCOSX),[11] with the appropriate auxiliary basis sets.[12] 'Tight' optimisation criteria (10^{-8} Ha tolerance for SCF, 10^{-6} Ha tolerance for optimisation step) were employed along with Grid6 / GridX6, corresponding to a Lebedev-590 angular grid, and a radial integral accuracy (IntAcc) of 5.34. Stationary points for the model systems were characterised through calculation of the Hessian. Minima were characterised by the absence of imaginary frequencies, and TSs by the presence of a single imaginary mode. Grimme's quasi-RRHO method[13] was used to calculate entropic corrections to obtain free energies at 298.15 K as implemented in the Python package *otherm*.[14] For reactions calculated in the gas phase, a 1 atm standard state was employed. For reactions in implicit solvent, a 1 atm to 1 M standard state correction was applied by adding $RT\ln(24.5) = 1.89$ kcal mol^{-1} to the calculated free energy of each species. NBO occupation numbers were calculated using the NBO program (v. 7.0), and ELF descriptors were calculated with Multiwfn (v. 3.6).[15]

All data processing was carried out using the Scikit-learn package in Python 3.7,[16] and MLR plots were generated with Matplotlib.[17] A Python script to generate plots is included as part of the Supporting Information. Individual figures can be generated interactively, or to plot all figures using the terminal run:

```
for i in {1..21}; do echo $i | python mlr_models.py -v; done
```

Enthalpies were chosen for a direct comparison with strain energies, which are commonly reported instead of Gibbs free energies. Trends in enthalpy and Gibbs free energy were found to be in excellent agreement for all reactions studied here. Values of $2 - N_{occ}$ were found to be in good agreement with an alternative density-based delocalization parameter, $\frac{D_\sigma}{D_\sigma^0}$, which was evaluated at the bond critical point (Fig. S3).[18]

S2 Linear free energy and geometric relationships

Activation barrier prediction

The Marcus equation for a chemical reaction is given by eq S1, where $\Delta E^{\ddagger}$ is the activation barrier for a given reaction and $\Delta E^{\ddagger}_{int}$ is the intrinsic activation barrier in the absence of a driving force ($\Delta E_r = 0$).

$$\Delta E^{\ddagger} = \Delta E^{\ddagger}_{int} + \frac{1}{2}\Delta E_r + \frac{\Delta E_r^2}{16\Delta E^{\ddagger}_{int}} \tag{S1}$$

For two similar reactions with equal driving force, according to eq S1 any difference in their activation barriers will be described by the difference in their intrinsic activation barriers, $\Delta\Delta E^{\ddagger}_{int}$:

$$\Delta\Delta E^{\ddagger} = \Delta\Delta E^{\ddagger}_{int} \tag{S2}$$

Combining equations S1 and S2, we arrive at a variant of the Marcus model that accounts for the effect of varying both the reaction driving force (through ΔE_r) and the intrinsic activation barrier (through $\Delta E^{\ddagger}_{int}$) on the observed activation energy (eq S3), relative to a reference intrinsic reaction barrier $\Delta E^{\ddagger}_{int}(0)$, where $\Delta E^{\ddagger}_{int} = \Delta E^{\ddagger}_{int}(0) + \Delta\Delta E^{\ddagger}_{int}$. The sensitivity of $\Delta E^{\ddagger}$ toward variation in the driving force and intrinsic activation barrier are given by equations S4a and S4b, respectively.

$$\Delta E^{\ddagger} = \Delta E^{\ddagger}_{int}(0) + \frac{1}{2}\Delta E_r + \frac{1}{16}\frac{\Delta E_r^2}{\Delta E^{\ddagger}_{int}(0) + \Delta\Delta E^{\ddagger}_{int}} + \Delta\Delta E^{\ddagger}_{int} \tag{S3}$$

$$\left(\frac{\partial \Delta E^{\ddagger}}{\partial \Delta E_r}\right)_{\Delta\Delta E^{\ddagger}_{int}} = \frac{1}{2} + \frac{1}{8}\left(\frac{\Delta E_r}{\Delta E^{\ddagger}_{int}(0) + \Delta\Delta E^{\ddagger}_{int}}\right) \tag{S4a}$$

$$\left(\frac{\partial \Delta E^{\ddagger}}{\partial \Delta\Delta E^{\ddagger}_{int}}\right)_{\Delta E_r} = 1 - \frac{1}{16}\left(\frac{\Delta E_r}{\Delta E^{\ddagger}_{int}(0) + \Delta\Delta E^{\ddagger}_{int}}\right)^2 \tag{S4b}$$

While the value of $\Delta\Delta E^{\ddagger}_{int}$ is a priori unknown, we can replace it with a calculated parameter that captures the physical origin of the change in $\Delta E^{\ddagger}_{int}$ for the range of systems of interest. For example, in the context of small ring reactivity, we propose that a more delocalized bond will be associated with a lower intrinsic activation barrier; in other words, it will be inherently easier to break a more delocalized bond. We can formulate a linear free energy relationship (eq S5) based on this hypothesis in which we use the parameterization

$\Delta\Delta E^{\ddagger}_{int} = \kappa\chi$, where χ is a variable that captures bond delocalization (vide infra) and κ is a proportionality constant that gives $\kappa\chi$ the units of energy. The sensitivity constants defined in equations S4a and S4b are replaced by fitting parameters α and β, respectively, where κ has been absorbed into the β parameter. An increase in driving force is expected to decrease $\Delta E^{\ddagger}$ ($\alpha > 0$), and an increase in bond delocalization is also predicted to decrease $\Delta E^{\ddagger}$ ($\beta > 0$). These parameters may be found by MLR using a Bell-Evans-Polanyi-type approximation, through which ΔE_r and $\Delta\Delta E^{\ddagger}_{int}$ are assumed to be uncorrelated. The quality of this assumption can be assessed through deviation of α and β from $\frac{1}{2}$ and κ, respectively.

$$\Delta E^{\ddagger} = \Delta E^{\ddagger}_{int}(0) + \alpha\Delta E_r + \beta\chi \tag{S5}$$

Transition state geometry prediction

We may derive an equation to predict the extension of a breaking bond from its equilibrium value to that found at the TS as follows:

The equations of two parabolas, E_1 and E_2, that describe the diabatic potential energy surfaces of a reaction are given by

$$E_1 = \frac{1}{2}kr^2 \tag{S6a}$$

$$E_2 = \frac{1}{2}k(r - r')^2 + \Delta E_r \tag{S6b}$$

where k is the force constant associated with the stretch of each bond, r is the length of the breaking bond, r' is the hypothetical length at which the breaking bond is fully cleaved, and ΔE_r is the reaction driving force. The x coordinate of the intersection point of the two parabolas ($\Delta r^{\ddagger}$), representing the position of the TS, is given by

$$\Delta r^{\ddagger} = \frac{1}{2}r' + \frac{\Delta E_r}{kr'} \tag{S7}$$

Using a Marcus-type approach, we can define $\frac{1}{2}r'$ as the intrinsic TS bond extension ($\Delta r^{\ddagger}_{int}$), for a symmetrical TS in which $\Delta E_r = 0$ such that eq S7 becomes

$$\Delta r^{\ddagger} = \Delta r^{\ddagger}_{int} + \frac{\Delta E_r}{2k\Delta r^{\ddagger}_{int}} \tag{S8}$$

This equation represents a Bell-Evans-Polanyi-like equation for the prediction of TS geometric parameters using knowledge of only the reaction driving force, the force constant associated with the breaking bond, and the intrinsic bond extension.

To enable examination of the effect of varying $\Delta r^{\ddagger}_{int}$ on $\Delta r^{\ddagger}$, we may use the substitution $\Delta r^{\ddagger}_{int} = \Delta r^{\ddagger}_{int}(0) + \Delta\Delta r^{\ddagger}_{int}$, resulting in eq S9, where $\Delta r^{\ddagger}_{int}(0)$ is the intrinsic TS bond extension for $\Delta E_r = 0$ and $\Delta\Delta r^{\ddagger}_{int} = 0$. Partial derivatives with respect to ΔE_r (fixed $\Delta r^{\ddagger}_{int}$) and $\Delta r^{\ddagger}_{int}$ (fixed ΔE_r) give the sensitivity of $\Delta r^{\ddagger}$ toward changes in driving force (eq S10a) and intrinsic bond extension (eq S10b), respectively.

$$\Delta r^{\ddagger} = \Delta r^{\ddagger}_{int}(0) + \frac{1}{2k}\frac{\Delta E_r}{\Delta r^{\ddagger}_{int}(0) + \Delta\Delta r^{\ddagger}_{int}} + \Delta\Delta r^{\ddagger}_{int} \tag{S9}$$

$$\left(\frac{\partial \Delta r^{\ddagger}}{\partial \Delta E_r}\right)_{\Delta\Delta r^{\ddagger}_{int}} = \frac{1}{2k}\left(\frac{1}{\Delta r^{\ddagger}_{int}(0) + \Delta\Delta r^{\ddagger}_{int}}\right) \tag{S10a}$$

$$\left(\frac{\partial \Delta r^{\ddagger}}{\partial \Delta\Delta r^{\ddagger}_{int}}\right)_{\Delta E_r} = 1 - \frac{1}{2k}\left(\frac{\Delta E_r}{(\Delta r^{\ddagger}_{int}(0) + \Delta\Delta r^{\ddagger}_{int})^2}\right) \tag{S10b}$$

Since $\Delta\Delta r^{\ddagger}_{int}$ is *a priori* unknown, we can replace it with a calculated parameter, in the same way as for the prediction of TS barriers in eq S5. For example, to investigate the role of bond delocalization on TS geometry, we can formulate a linear geometric relationship, first using the parameterization $\Delta\Delta r^{\ddagger}_{int} = \lambda\chi$, where χ has the same meaning as before, and λ is a proportionality constant that gives $\lambda\chi$ the units of distance. Second, the sensitivity constants defined in equations S10a and S10b may be replaced by fitting parameters γ and δ, respectively, where λ has been absorbed into the δ parameter. An increase in driving force is expected to decrease $\Delta r^{\ddagger}$ ($\gamma > 0$), and an increase in bond delocalization is also predicted to decrease $\Delta r^{\ddagger}$ ($\delta > 0$). As before, we may find these parameters using MLR, where we again make a Bell-Evans-Polanyi-type approximation through which ΔE_r and $\Delta\Delta r^{\ddagger}_{int}$ are assumed to be uncorrelated. The quality of this assumption can be assessed through deviation of γ and δ from $(2k\Delta r^{\ddagger}_{int}(0))^{-1}$ and λ, respectively.

$$\Delta r^{\ddagger} = \Delta r^{\ddagger}_{int}(0) + \gamma\Delta E_r + \delta\chi \tag{S11}$$

A value of $k = 474.4$ kcal mol^{-1} Å^{-2} is obtained from a plot of $\Delta H^{\ddagger}$ vs $(\Delta r^{\ddagger})^2$, and assuming $\Delta r^{\ddagger}_{int}(0) = 0.438$ Åfor the intrinsic bond extension of ethane, a value of $(2k\Delta r^{\ddagger}_{int}(0))^{-1} = 0.0024$ mol kcal^{-1} is obtained – in satisfactory agreement with the value of γ (0.0017 mol kcal^{-1}) from MLR.

S3 Hydrocarbon ring-opening reactivity

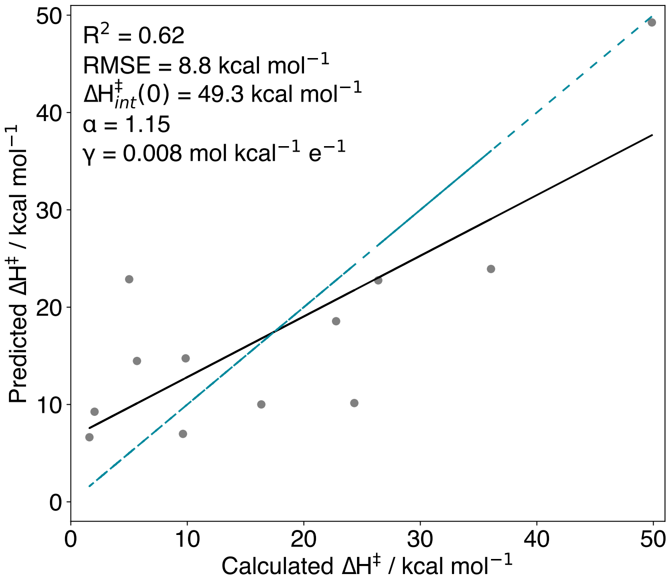

Figure S1: MLR plot ($CH_3^{\bullet}$ + hydrocarbon) for the prediction of $\Delta H^{\ddagger}$ from ΔH_r and ΔH_r^2 (Marcus) using $\Delta H^{\ddagger} = \Delta H^{\ddagger}_{\text{int}} + \alpha \Delta H_r + \beta \Delta H_r^2$, where α and β are optimised coefficients. The blue dashed line denotes perfect correlation.

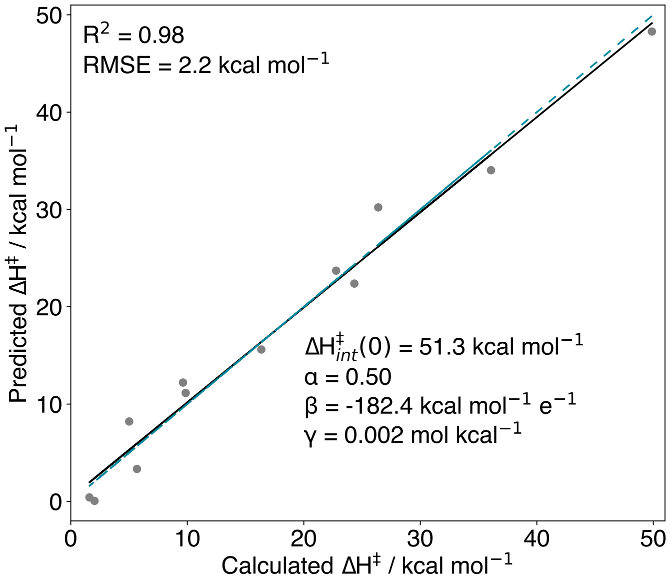

Figure S2: MLR plot ($CH_3^{\bullet}$ + hydrocarbon) for the prediction of $\Delta H^{\ddagger}$ from ΔH_r, ΔH_r^2 and $2 - N_{occ}$ (Marcus + delocalization) using $\Delta H^{\ddagger} = \Delta H^{\ddagger}_{\text{int}} + \alpha \Delta H_r + \beta(2 - N_{occ}) + \gamma \Delta H_r^2$, where α, β and γ are optimised coefficients. The blue dashed line denotes perfect correlation.

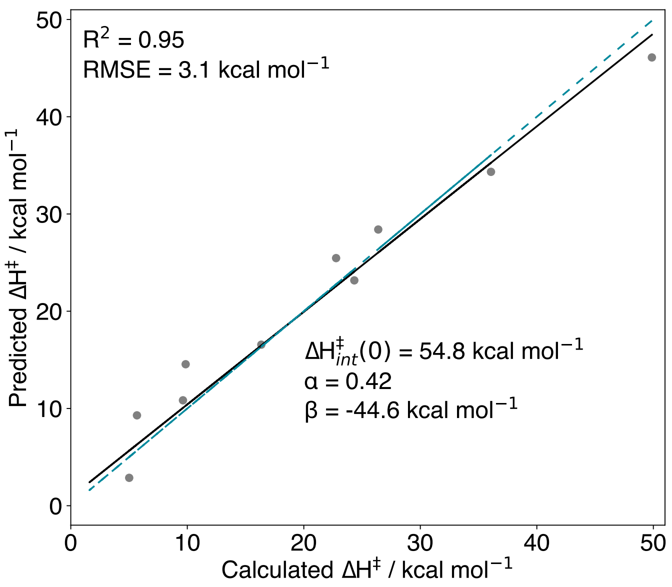

Figure S3: MLR plot ($CH_3^{\bullet}$ + hydrocarbon) for the prediction of $\Delta H^{\ddagger}$ from ΔH_r and $\frac{D_\sigma}{D_\sigma^0}$ using $\Delta H^{\ddagger} = \Delta H^{\ddagger}_{\text{int}} + \alpha \Delta H_r + \beta \frac{D_\sigma}{D_\sigma^0}$, where α and β are optimised coefficients. The blue dashed line denotes perfect correlation.

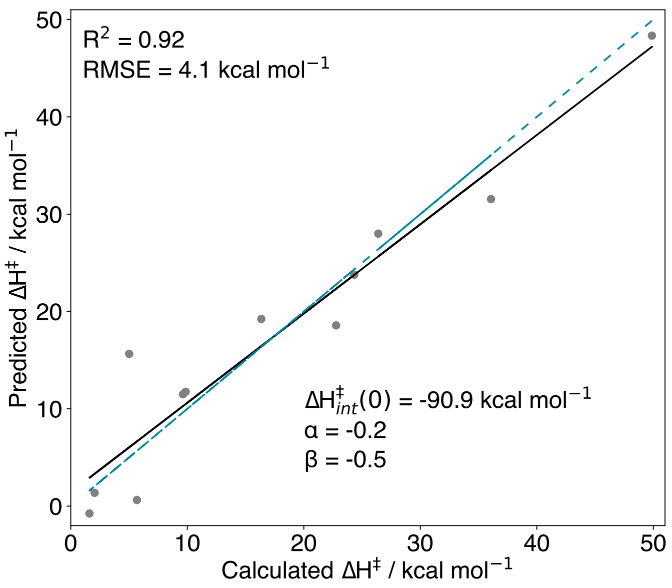

Figure S4: MLR plot ($CH_3^{\bullet}$ + hydrocarbon) for the prediction of $\Delta H^{\ddagger}$ from ΔH_r and E_{HOMO} using $\Delta H^{\ddagger} = \Delta H^{\ddagger}_{\text{int}} + \alpha \Delta H_r + \beta E_{HOMO}$, where α and β are optimised coefficients. The blue dashed line denotes perfect correlation.

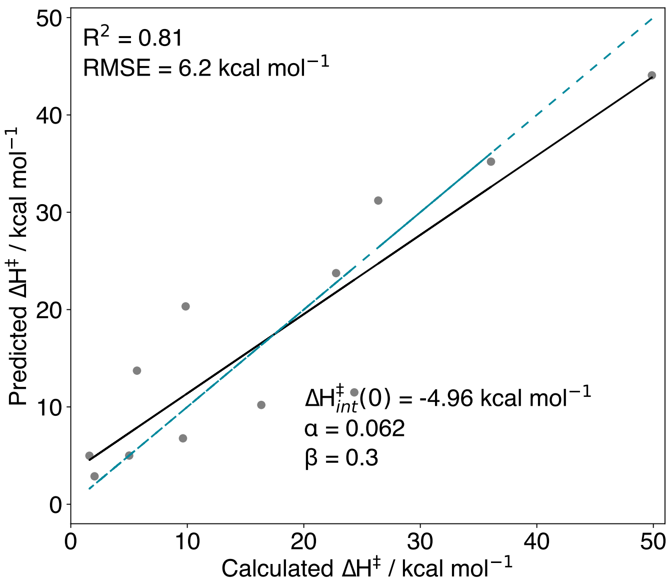

Figure S5: MLR plot ($CH_3^\bullet$ + hydrocarbon) for the prediction of $\Delta H^\ddagger$ from ΔH_r and E_{LUMO} using $\Delta H^\ddagger = \Delta H^\ddagger_{\text{int}} + \alpha \Delta H_r + \beta E_{LUMO}$, where α and β are optimised coefficients. The blue dashed line denotes perfect correlation.

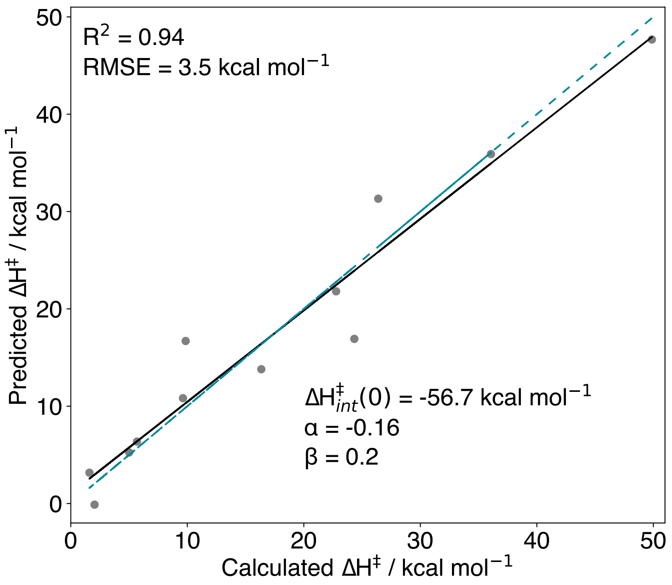

Figure S6: MLR plot ($CH_3^\bullet$ + hydrocarbon) for the prediction of $\Delta H^\ddagger$ from ΔH_r and $\Delta E_{HOMO-LUMO}$ using $\Delta H^\ddagger = \Delta H^\ddagger_{\text{int}} + \alpha \Delta H_r + \beta \Delta E_{HOMO-LUMO}$, where α and β are optimised coefficients. The blue dashed line denotes perfect correlation.

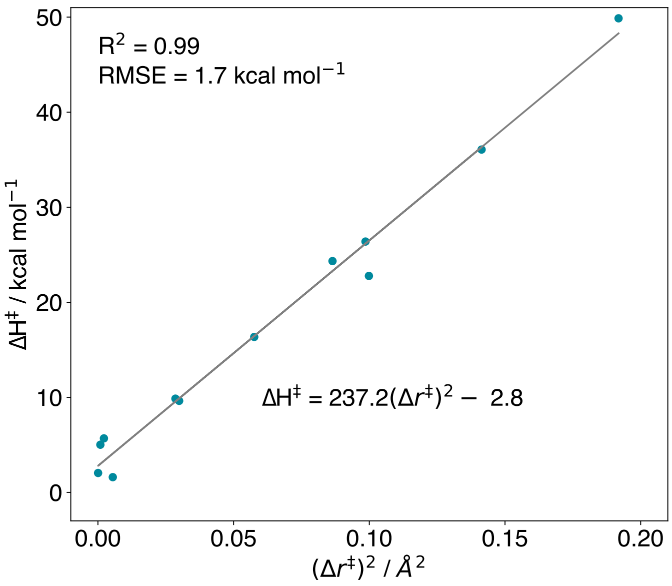

Figure S7: Prediction of $\Delta H^\ddagger$ from the TS bond extension, $\Delta r^\ddagger$, where $\Delta r^\ddagger = r^\ddagger - r_0$. Bond lengths in Å, energies in kcal mol^{-1}.

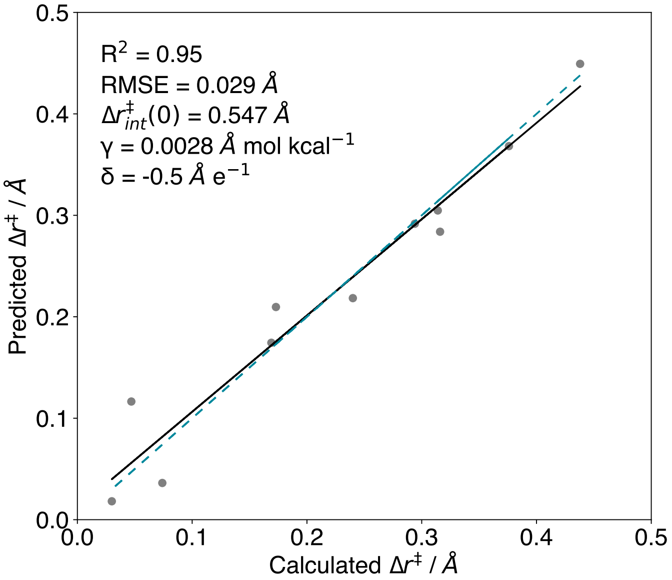

Figure S8: Prediction of $\Delta r^\ddagger$ from ΔH_r and $\frac{D_\sigma}{D_\sigma^0}$. The blue dashed line denotes perfect correlation.

Molecule	$\Delta H^{\ddagger}_{\text{calc}}$	$\Delta H^{\ddagger}_{\text{pred}}(\chi)$		
		$2 - N_{occ}$	D/D_0	n_3
Ethane (**A**)	49.9	45.7	46.1	49.9
Cyclopropane (**B**)	26.4	31.1	28.3	25.8
Cyclobutane (**C**)	36.1	35.0	34.3	37.0
Bicyclo[1.1.0]butane (**D**)	9.9	12.0	14.5	9.0
Bicyclo[2.1.0]pentane (**E**)	16.4	16.8	16.5	14.0
Bicyclo[3.1.0]hexane (**F**)	22.8	24.8	25.4	22.7
Bicyclo[2.2.0]hexane (**G**)	24.3	24.0	23.1	24.7
[1.1.1]Propellane (**H**)	5.0	7.9	2.8	4.8
[2.1.1]Propellane (**I**)	2.0	0.4	–	2.4
[3.1.1]Propellane (**J**)	5.7	3.7	9.2	8.7
[2.2.1]Propellane (**K**)	1.6	–0.8	–4.2	1.7
[2.2.2]Propellane (**L**)	9.6	11.2	10.7	10.5

Table S1: Calculated and predicted activation enthalpies (kcal mol^{-1}) for the addition of a methyl radical to each of the molecules in the H12 set. Enthalpies calculated at the DLPNO-CCSD(T)/def2-QZVPP (TightPNO) level, with thermal corrections from the B2PLYP-D3BJ/def2-TZVP level.

Molecule	$\Delta H^{\ddagger}_{\text{calc}}$	$\Delta H^{\ddagger}_{\text{pred}}(\chi)$		
		$2 - N_{occ}$	D/D_0	n_3
Ethane (**A**)	66.7	60.1	59.2	63.0
Cyclopropane (**B**)	40.6	44.8	41.8	39.3
Cyclobutane (**C**)	49.5	49.6	48.7	51.5
Bicyclo[1.1.0]butane (**D**)	21.4	23.6	27.0	21.5
Bicyclo[2.1.0]pentane (**E**)	28.6	29.9	30.6	28.3
Bicyclo[3.1.0]hexane (**F**)	38.7	38.6	39.6	37.1
Bicyclo[2.2.0]hexane (**G**)	34.6	39.0	39.2	41.0
[1.1.1]Propellane (**H**)	13.2	15.5	10.2	12.0
[2.1.1]Propellane (**I**)	7.5	6.6	–	8.4
[3.1.1]Propellane (**J**)	18.2	15.0	21.8	21.5
[2.2.1]Propellane (**K**)	6.6	4.4	1.8	6.6
[2.2.2]Propellane (**L**)	21.2	19.0	19.3	18.3

Table S2: Calculated and predicted activation enthalpies (kcal mol^{-1}) for the addition of amide anion NH_2^- to each of the molecules in the H12 set. Enthalpies calculated at the SMD(THF)-DLPNO-CCSD(T)/ma-def2-QZVPP (TightPNO) level, with thermal corrections from the SMD(THF)-B2PLYP-D3BJ/def2-TZVP (ma-def2-TZVP on N) level.

$CCl_3^{\bullet}$ addition		B2PLYP-D3BJ					DLPNO-CCSD(T)		
		ΔE_{el}	ΔZPE	ΔH	$T\Delta S$	ΔG	ΔE	ΔH	ΔG
[1.1.1]Propellane, **H**	vdW complex	-2.7	0.3	-1.5	-10.5	9.1	-1.6	-0.4	10.2
	TS	-1.0	0.3	-0.6	-12.2	11.6	0.1	0.5	12.8
	rxn	-25.8	2.2	-24.0	-13.8	-10.2	-26.6	-24.8	-11.0
Bicyclo[1.1.0]butane, **D**	vdW complex	-2.9	0.4	-1.7	-9.9	8.3	-2.2	-0.9	9.0
	TS	2.5	0.3	2.8	-12.2	15.0	3.6	4.0	16.2
	rxn	-40.3	1.4	-39.0	-12.9	-26.1	-41.4	-40.1	-27.2
Bicyclo[2.1.0]pentane, **E**	vdW complex	-3.3	0.4	-2.1	-10.6	8.5	-2.5	-1.3	9.3
	TS	8.8	-0.1	8.8	-12.4	21.3	10.6	10.7	23.2
	rxn	-51.3	1.6	-49.8	-13.1	-36.7	-51.7	-50.2	-37.1

Table S3: Differences in thermodynamic quantities (kcal mol^{-1}) for the addition of $CCl_3^{\bullet}$ to [1.1.1]propellane (**H**), bicyclo[1.1.0]butane (**D**) and bicyclo[2.1.0]pentane (**E**), optimised at the B2PLYP-D3BJ/def2-TZVP level, with single point energies calculated at the DLPNO-CCSD(T)/def2-QZVPP (TightPNO) level. ΔH and ΔG at the DLPNO-CCSD(T) level calculated using thermal corrections from the B2PLYP level.

$CH_3^{\bullet}$ addition		B2PLYP-D3BJ					DLPNO-CCSD(T)		
		ΔE_{el}	ΔZPE	ΔH	$T\Delta S$	ΔG	ΔE	ΔH	ΔG
Ethane, **A**	TS	49.2	1.2	49.5	-10.4	59.9	49.7	49.9	60.3
	rxn	0.0	0.0	0.0	0.0	0.0	0.0	0.0	0.0
Cyclopropane, **B**	TS	25.2	1.6	25.9	-10.5	36.5	25.7	26.4	36.9
	rxn	-31.2	3.8	-28.3	-10.7	-17.7	-31.2	-28.4	-17.7
Cyclobutane, **C**	TS	35.1	1.6	35.7	-11.0	46.6	35.5	36.1	47.0
	rxn	-30.1	3.1	-27.5	-9.8	-17.7	-29.4	-26.8	-16.9
Bicyclo[1.1.0]butane, **D**	TS	7.8	1.9	8.9	-10.5	19.4	8.8	9.9	20.4
	rxn	-45.2	4.6	-41.8	-11.2	-30.6	-45.0	-41.6	-30.3
Bicyclo[2.1.0]pentane, **E**	TS	14.4	1.6	15.3	-10.6	25.9	15.5	16.4	27.0
	rxn	-57.3	4.8	-53.6	-11.4	-42.2	-56.5	-52.9	-41.4
Bicyclo[3.1.0]hexane, **F**	TS	21.0	2.0	22.0	-11.3	33.4	21.7	22.8	34.1
	rxn	-38.9	4.8	-35.3	15.4	-23.5	-38.3	-34.7	-23.0
Bicyclo[2.2.0]hexane, **G**	TS	22.8	1.8	23.6	-11.1	34.8	23.5	24.3	35.5
	rxn	-57.9	5.5	-53.7	-11.9	-41.8	-56.7	-52.4	-40.5
[1.1.1]Propellane, **H**	TS	2.9	1.7	3.9	-10.3	14.1	4.0	5.0	15.3
	rxn	-32.4	5.5	-28.4	-12.2	-16.2	-32.2	-28.2	-16.0
[2.1.1]Propellane, **I**	TS	0.3	1.4	1.2	-9.9	11.1	1.1	2.0	12.0
	rxn	-59.7	6.2	-55.1	-12.6	-42.5	-59.8	-55.3	-42.7
[3.1.1]Propellane, **J**	TS	3.4	1.8	4.4	-10.7	15.2	4.7	5.7	16.4
	rxn	-46.2	5.9	-41.9	-12.4	-29.5	-46.4	-42.1	-29.7
[2.2.1]Propellane, **K**	TS	-0.4	1.4	0.5	-10.2	10.6	0.7	1.6	11.8
	rxn	-83.5	7.1	-78.2	-12.9	-65.3	-83.8	-78.5	-65.6
[2.2.2]Propellane, **L**	TS	7.2	1.8	8.1	-11.1	19.3	8.6	9.6	20.8
	rxn	-88.6	7.8	-82.5	-13.1	-69.4	-88.1	-82.1	-69.0

Table S4: Differences in thermodynamic quantities (kcal mol^{-1}) for the addition of a methyl radical to each of the molecules in the H12 set, optimised at the B2PLYP-D3BJ/def2-TZVP level, with single point energies calculated at the DLPNO-CCSD(T)/def2-QZVPP (TightPNO) level. ΔH and ΔG at the DLPNO-CCSD(T) level calculated using thermal corrections from the B2PLYP level.

NH_2^- addition		B2PLYP-D3BJ					DLPNO-CCSD(T)		
		ΔE_{el}	ΔZPE	ΔH	$T\Delta S$	ΔG	ΔE	ΔH	ΔG
Ethane, **A**	TS	66.2	-1.5	64.4	-8.1	72.5	68.5	66.7	74.8
	rxn	24.5	-0.2	24.3	0.8	23.5	22.4	22.3	21.4
Cyclopropane, **B**	TS	40.1	-0.3	39.3	-8.8	48.2	41.3	40.6	49.4
	rxn	1.7	3.3	4.1	-9.9	14.0	-2.2	0.2	10.0
Cyclobutane, **C**	TS	48.4	-0.2	47.5	-9.6	57.1	50.3	49.5	59.0
	rxn	3.8	2.8	6.0	-9.3	15.2	1.0	3.2	12.4
Bicyclo[1.1.0]butane, **D**	TS	19.7	1.2	20.2	-9.4	29.6	20.9	21.4	30.7
	rxn	-11.1	4.6	-8.0	-11.1	3.1	-15.2	-12.1	-1.0
Bicyclo[2.1.0]pentane, **E**	TS	27.2	-0.1	26.8	-9.0	35.8	29.0	28.6	37.6
	rxn	-18.4	4.4	-15.4	-11.0	-4.4	-21.2	-18.2	-7.2
Bicyclo[3.1.0]hexane, **F**	TS	36.4	-0.2	35.7	-9.4	45.1	39.3	38.7	48.0
	rxn	-3.5	4.0	-0.9	-11.5	10.6	-6.1	-3.5	8.0
Bicyclo[2.2.0]hexane, **G**	TS	32.6	0.4	32.4	-9.7	42.0	34.8	34.6	44.3
	rxn	-14.9	4.4	-11.7	-10.9	-0.8	-17.5	-14.3	-3.4
[1.1.1]Propellane, **H**	TS	12.1	1.0	12.4	-9.5	21.9	12.9	13.2	22.7
	rxn	-9.0	3.9	-6.5	-11.3	4.7	-13.1	-10.7	0.6
[2.1.1]Propellane, **I**	TS	6.5	1.0	6.9	-9.4	16.3	7.0	7.5	16.8
	rxn	-32.7	4.7	-29.6	-11.7	-18.0	-37.2	-34.1	-22.4
[3.1.1]Propellane, **J**	TS	17.0	0.8	17.1	-9.8	26.9	18.1	18.2	28.1
	rxn	-10.4	4.1	-7.7	-11.3	3.6	-14.9	-12.2	-0.9
[2.2.1]Propellane, **K**	TS	5.2	1.0	5.8	-9.0	14.8	6.0	6.6	15.6
	rxn	-54.1	5.7	-50.2	-12.1	-38.1	-58.2	-54.3	-42.2
[2.2.2]Propellane, **L**	TS	19.0	0.7	19.1	-9.8	28.8	21.1	21.2	30.9
	rxn	-54.5	6.1	-50.1	-12.0	-38.1	-57.0	-52.6	-40.7

Table S5: Differences in thermodynamic quantities (kcal mol^{-1}) for the addition of an amide anion (NH_2^-) to each of the molecules in the H12 set, optimised at the SMD(THF)-B2PLYP-D3BJ/def2-TZVP (ma-def2-TZVP on N) level, with single point energies calculated at the SMD(THF)-DLPNO-CCSD(T)/ma-def2-QZVPP (TightPNO) level. ΔH and ΔG at the DLPNO-CCSD(T) level calculated using thermal corrections from the B2PLYP level.

S4 Heterocycle ring-opening reactivity

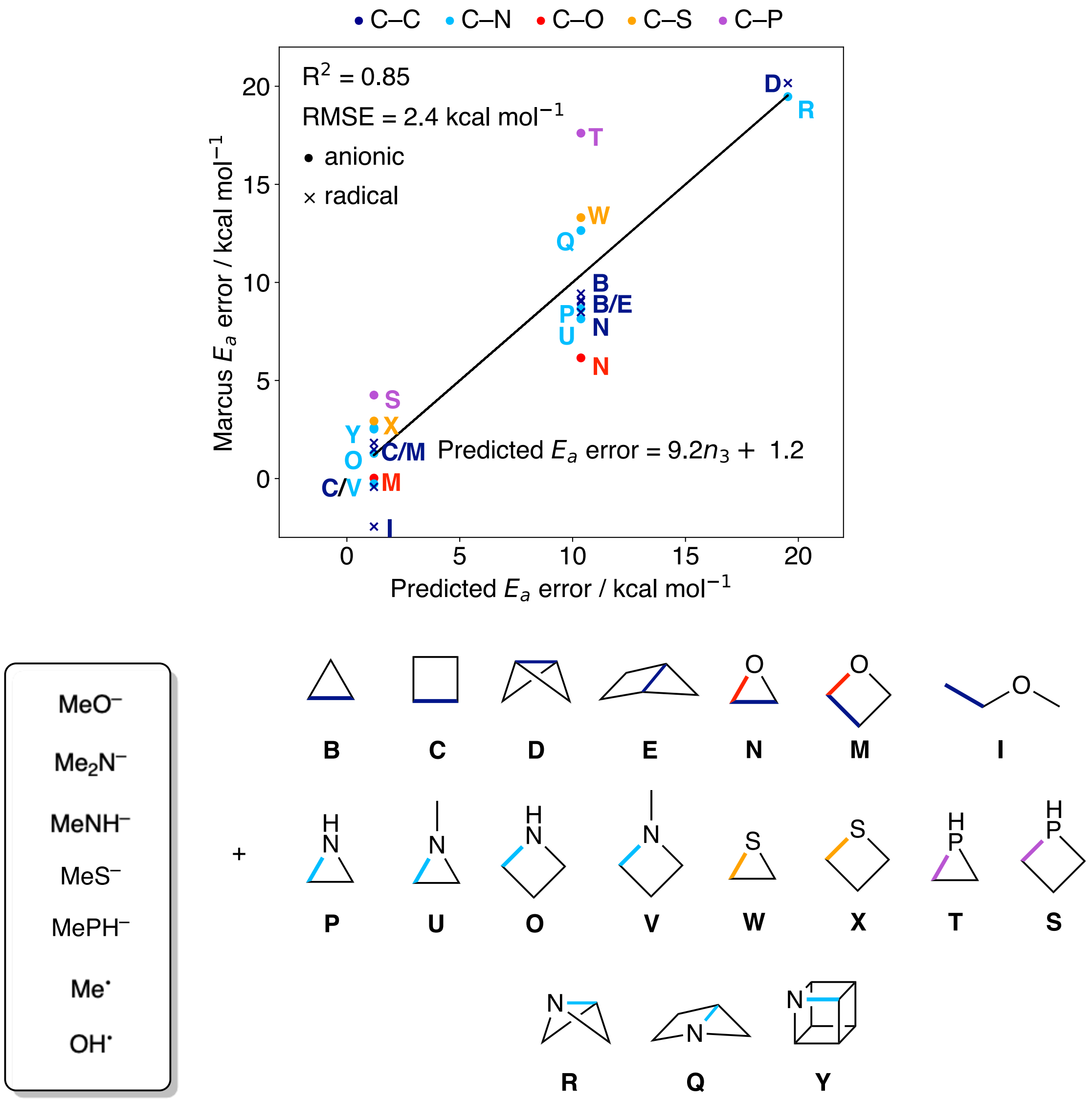

Figure S9: Anionic and radical additions to a set of 18 heterocyclic molecules (Fig. S10, and the error in activation energy (E_a) predicted by Marcus theory vs $2 - N_{occ}$. Reaction data from refs. [19, 20].

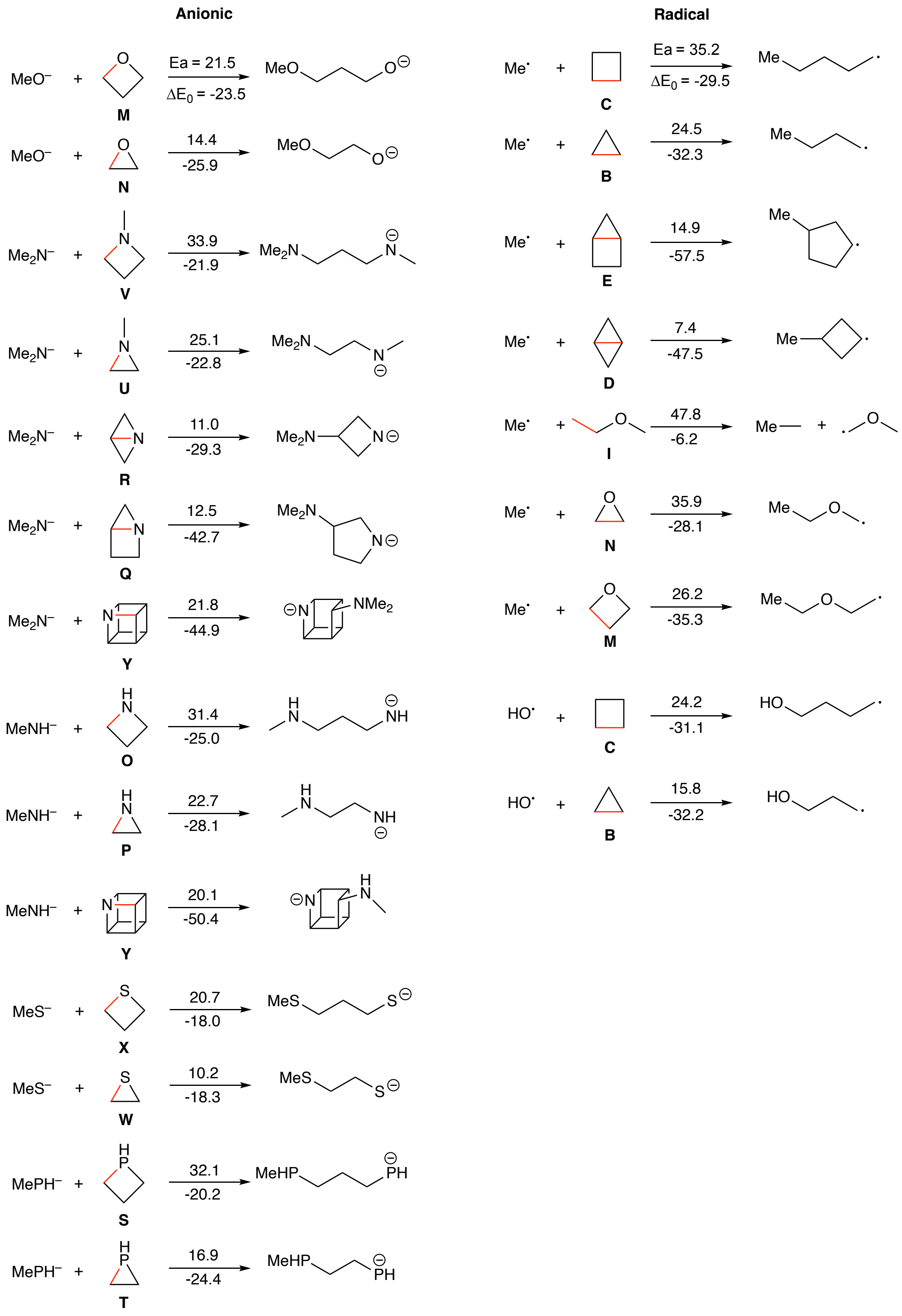

Figure S10: Anionic and radical reactions used to generate Fig. S9, with data taken from refs. [19, 20].

S5 Tabulated strain release energies and delocalization values

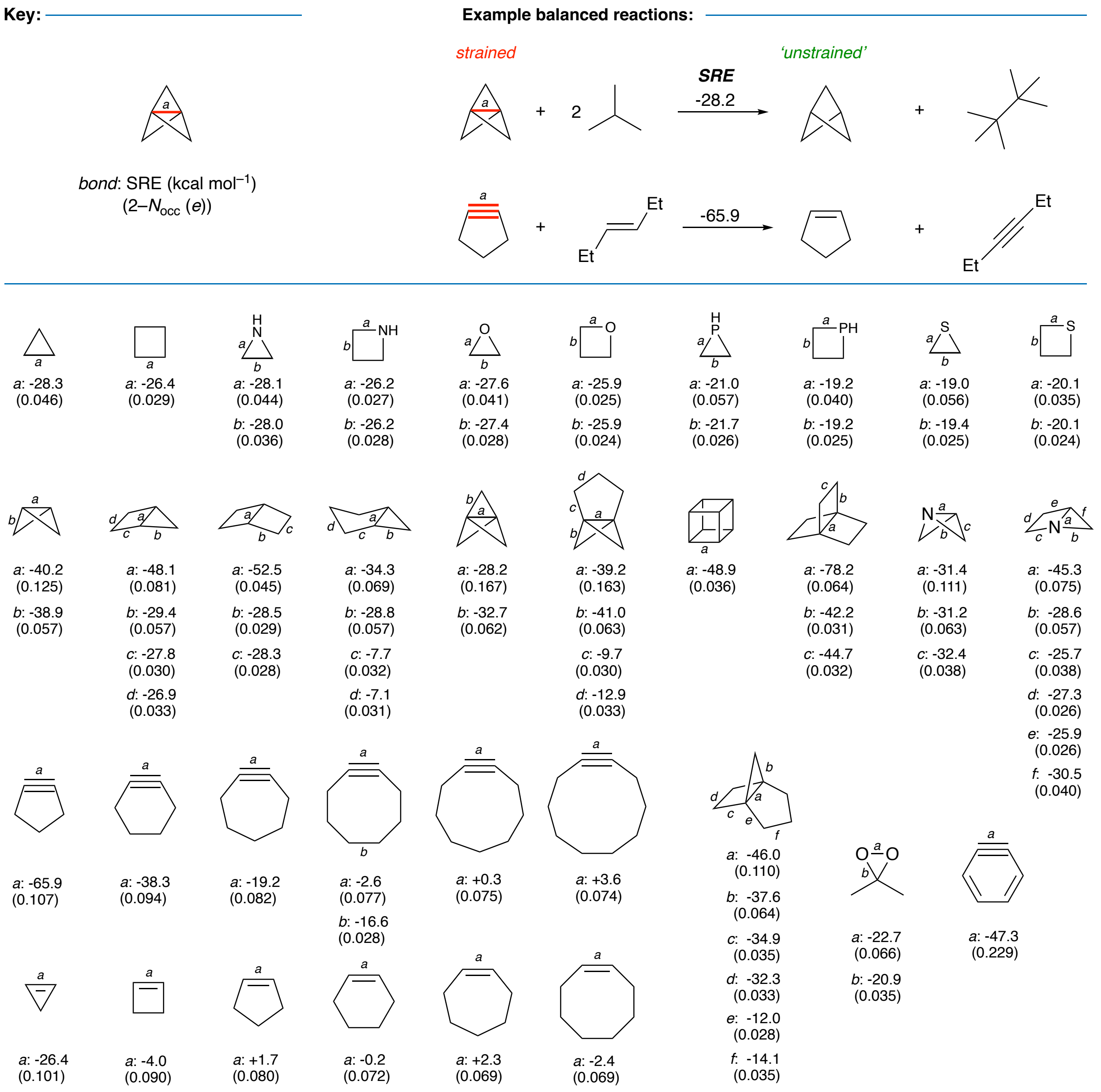

Figure S11: Set of strain release energies (SREs, kcal mol^{-1}) and $2 - N_{occ}$ values (e) per bond type for a range of mono-, bi- and tricyclic ring systems, cyclic alkynes and alkenes.

S6 'Rule of thumb' worked example

Shown in Fig. 5d, and reproduced here, is the addition of Bn_2NH to a sulfonyl bicyclo[1.1.0]butane (**D′**) or bicyclo[2.1.0]pentane (**E′**). While the former reaction takes place at 25 °C (298 K), the latter requires heating to 80 °C (353 K).

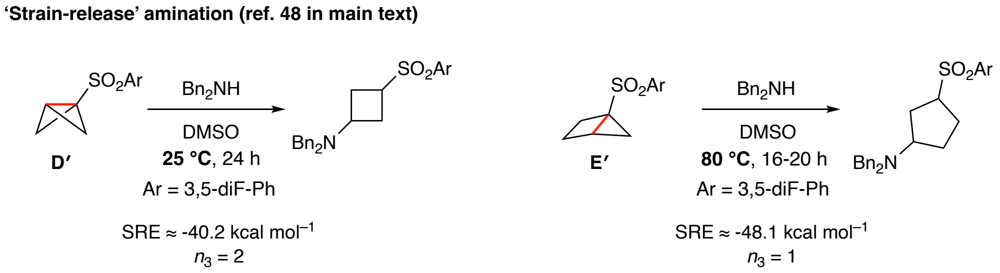

Using the 'rule of thumb' of $\Delta\Delta H^{\ddagger} \approx 0.5\Delta SRE - 10\Delta n_3$, for these reactions $\Delta SRE = -7.9$ kcal mol^{-1} and $\Delta n_3 = -1$. Based on strain release alone, **E′** should have an activation enthalpy ≈ 4 kcal mol^{-1} lower than **D′** – therefore a reaction rate ≈ 10^3 times greater than **D′** at 298 K. However, barrier lowering due to three-membered ring delocalization should independently lower the intrinsic barrier for **D′** by ≈ 10 kcal mol^{-1} relative to **E′**. The net effect is a prediction of a ≈ <u>6 kcal mol^{-1}</u> lower enthalpic barrier to the reaction for **D′** than **E′**, with delocalization overturning the strain release bias.

Based on the experimental data, we can roughly estimate the relative reaction rates for the two reactions at a given temperature ($k_{rel} = k_{D'}(T)/k_{E'}(T)$) using the Eyring equation, if we assume that the experimental conditions are identical except for changes in temperature, that the two mechanisms are identical, and that the reactions occur at identical rates at the two different temperatures used in the reactions (*i.e.*, $k_{D'}(T)/k_{E'}(T') = 1$).

Using the latter condition, the relationship between the free energy barriers of the two reactions and the two reaction temperatures is

$$\Delta G^{\ddagger}_{E'} = \frac{T'}{T}\Delta G^{\ddagger}_{D'} + RT' \ln \frac{T'}{T}$$

where R is the gas constant. Using $T'/T = 1.2$, we obtain $\Delta G^{\ddagger}_{E'} = 1.2\Delta G^{\ddagger}_{D'} + 0.1$ kcal mol^{-1}. Based on the reported reaction time of 24 h for **D′** at 298 K, we can estimate a value of $\Delta G^{\ddagger}_{D'} \approx 24$ kcal mol^{-1} ($t_{1/2} = 12$ h). The resulting estimate for $\Delta G^{\ddagger}_{E'}$ is then 29 kcal mol^{-1} – therefore the free energy barrier is ≈ <u>5 kcal mol^{-1}</u> lower for **D′** than **E′**, a difference of only 1 kcal mol^{-1} from the rule of thumb prediction. Despite completely neglecting entropic effects and avoiding any electronic structure calculations or experiments, the rule of thumb gives a good estimate of the expected reactivity difference based only on tabulated data and visual inspection.

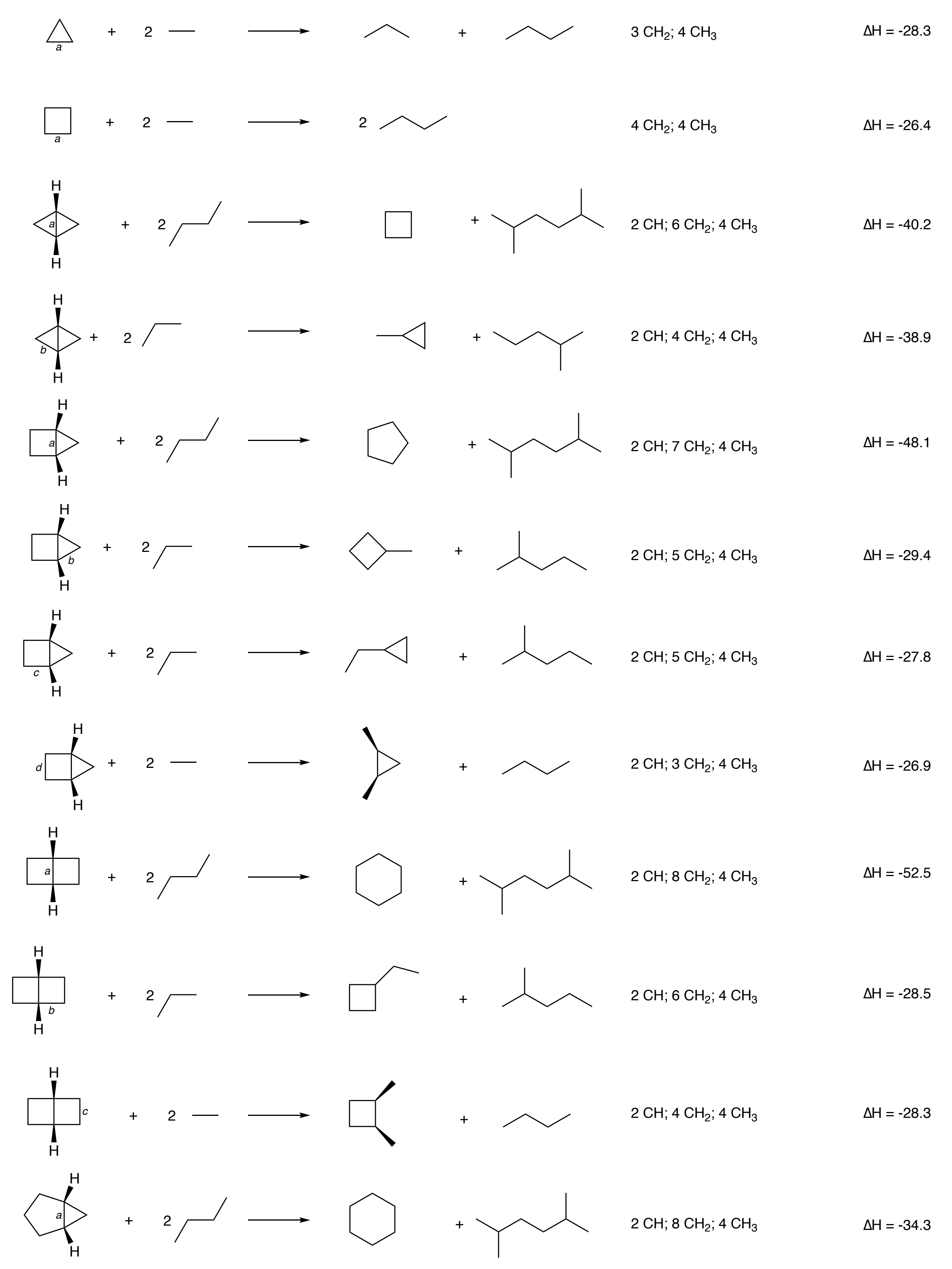

Figure S12: Balanced hydrogen transfer reactions by bond type, group classifications and reaction enthalpies (kcal mol^{-1}) calculated at the DLPNO-CCSD(T)/def2-QZVPP (TightPNO)//B2PLYP-D3BJ/def2-TZVP level.

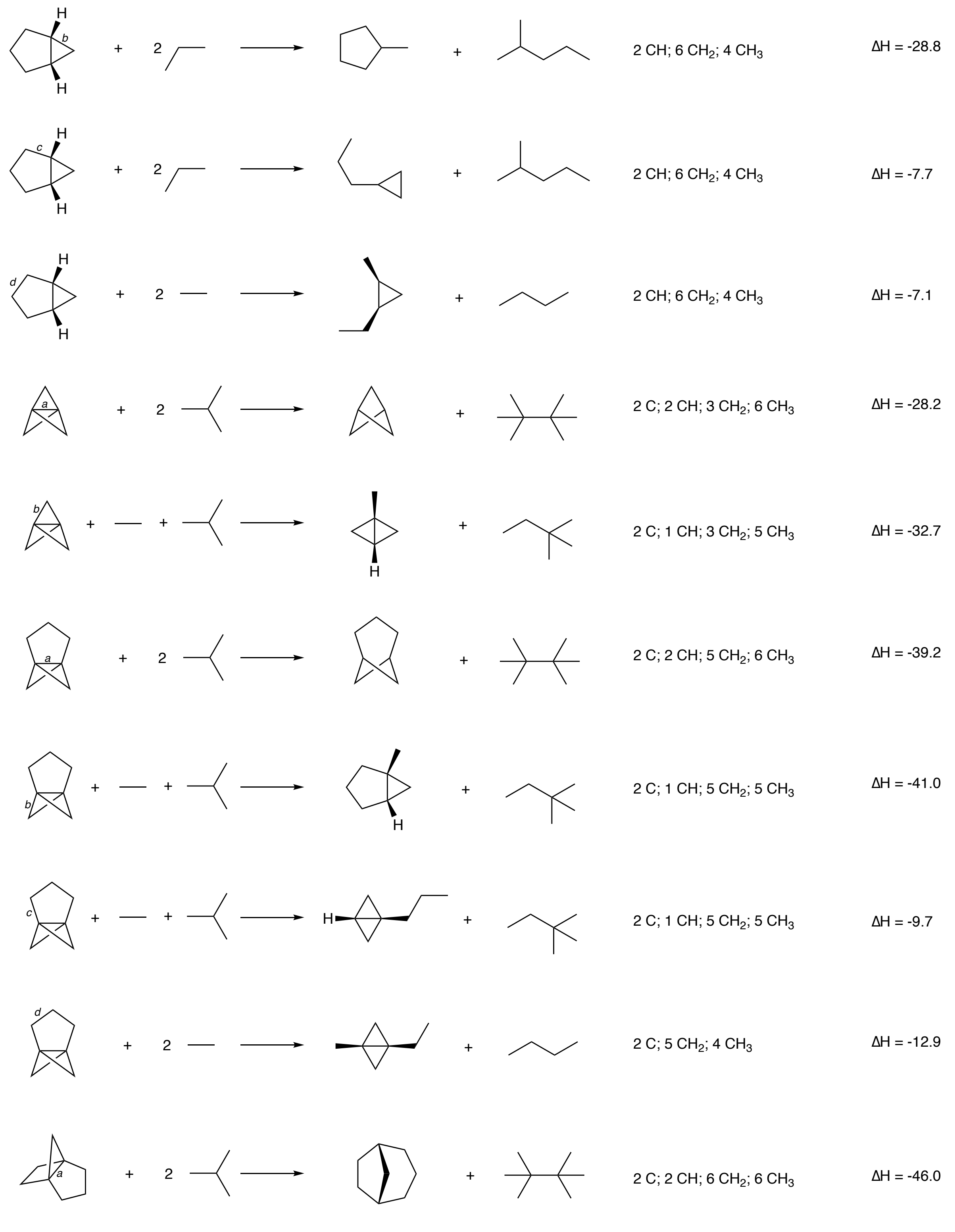

Figure S13: Balanced hydrogen transfer reactions by bond type, group classifications and reaction enthalpies (kcal mol^{-1}) calculated at the DLPNO-CCSD(T)/def2-QZVPP (TightPNO)//B2PLYP-D3BJ/def2-TZVP level.

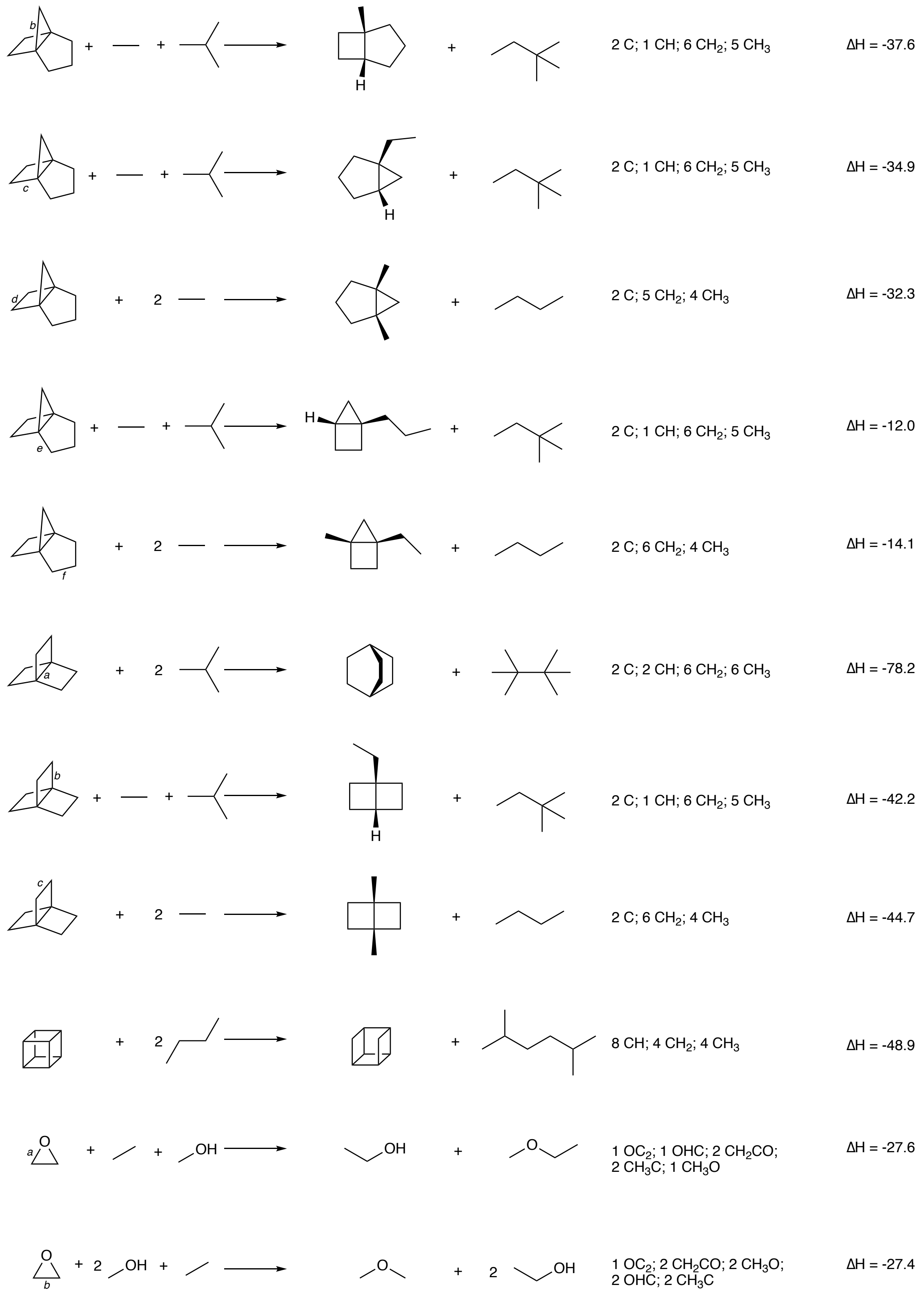

Figure S14: Balanced hydrogen transfer reactions by bond type, group classifications and reaction enthalpies (kcal mol^{-1}) calculated at the DLPNO-CCSD(T)/def2-QZVPP (TightPNO)//B2PLYP-D3BJ/def2-TZVP level.

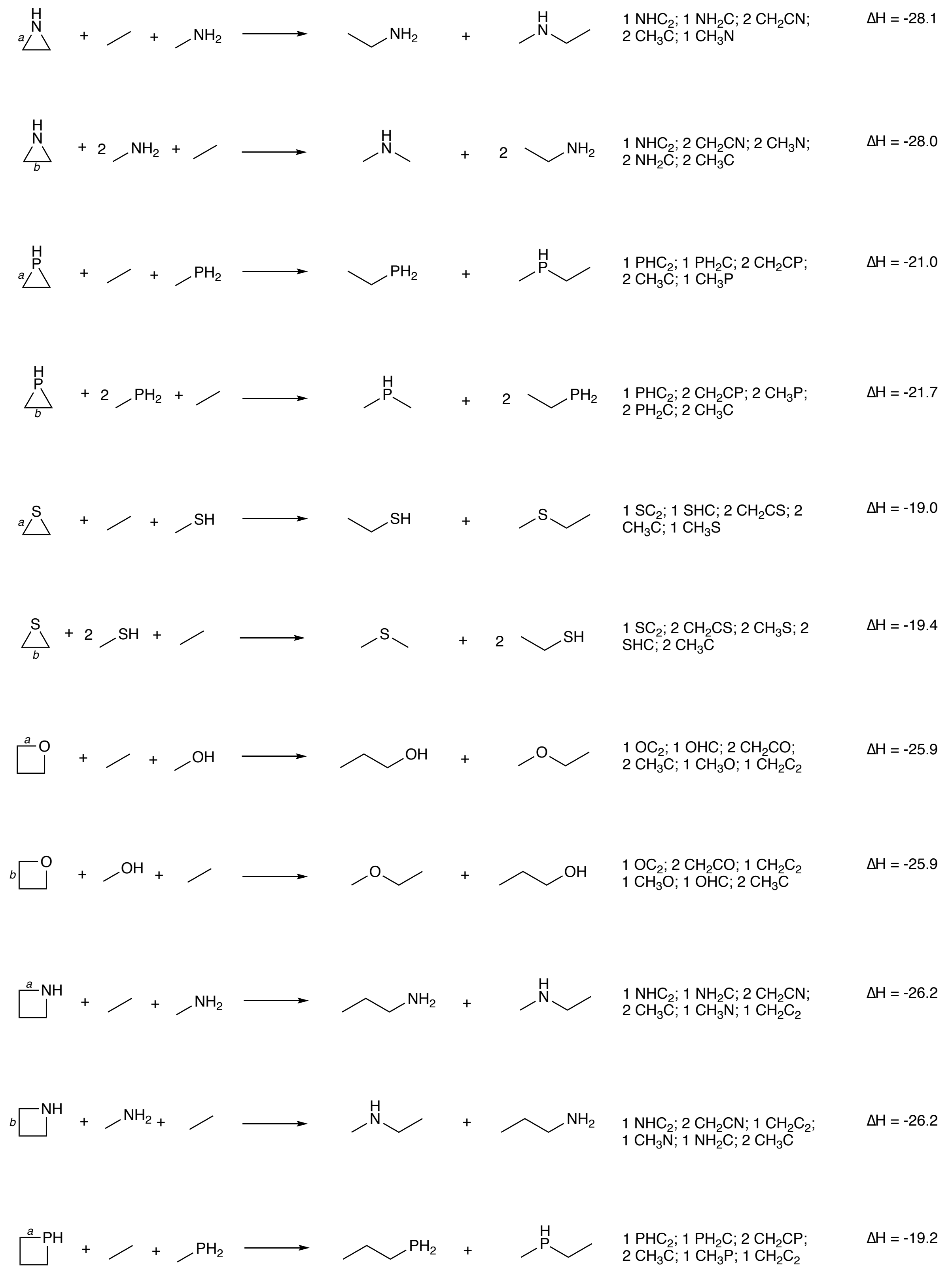

Figure S15: Balanced hydrogen transfer reactions by bond type, group classifications and reaction enthalpies (kcal mol^{-1}) calculated at the DLPNO-CCSD(T)/def2-QZVPP (TightPNO)//B2PLYP-D3BJ/def2-TZVP level.

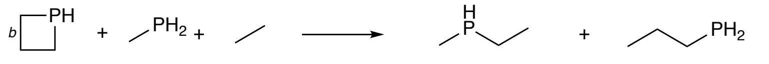

1 PHC_2; 2 CH_2CP; 1 CH_2C_2; 1 CH_3P; 1 PH_2C; 2 CH_3C — ΔH = -19.2

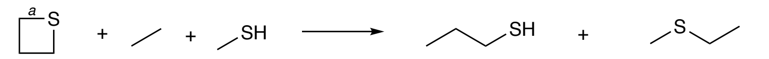

1 SC_2; 1 SHC; 2 CH_2CS; 2 CH_3C; 1 CH_3S; 1 CH_2C_2 — ΔH = -20.1

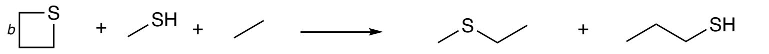

1 SC_2; 2 CH_2CS; 1 CH_2C_2; 1 CH_3S; 1 SHC; 2 CH_3C — ΔH = -20.1

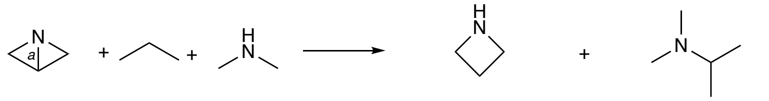

1 NC_3; 2 CH_2CN; 1 CHC_2N; 2 CH_3C; 1 CH_2C_2; 1 NHC_2; 2 CH_3N — ΔH = -31.4

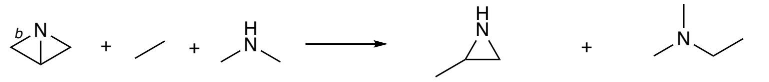

1 NC_3; 2 CH_2CN; 1 CHC_2N; 2 CH_3C; 1 NHC_2; 2 CH_3N — ΔH = -31.2

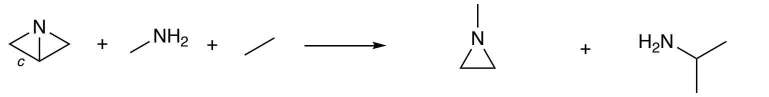

1 NC_3; 2 CH_2CN; 1 CHC_2N; 2 CH_3C; 1 NH_2C; 1 CH_3N — ΔH = -32.4

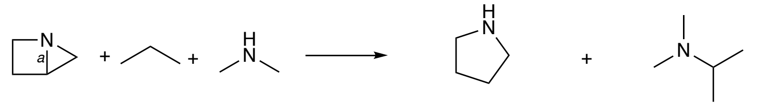

1 NC_3; 2 CH_2CN; 1 CHC_2N; 2 CH_3C; 2 CH_2C_2; 1 NHC_2; 2 CH_3N — ΔH = -45.3

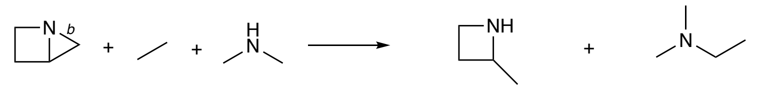

1 NC_3; 2 CH_2CN; 1 CHC_2N; 2 CH_3C; 1 NHC_2; 2 CH_3N; 1 CH_2C_2 — ΔH = -28.6

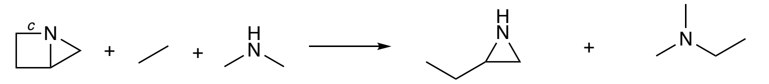

1 NC_3; 2 CH_2CN; 1 CHC_2N; 2 CH_3C; 1 NHC_2; 2 CH_3N; 1 CH_2C_2 — ΔH = -25.7

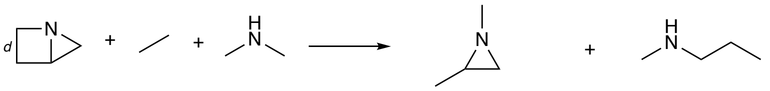

1 NC_3; 2 CH_2CN; 1 CHC_2N; 2 CH_3C; 1 NHC_2; 2 CH_3N; 1 CH_2C_2 — ΔH = -27.3

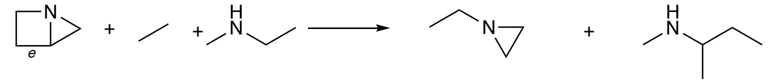

1 NC_3; 3 CH_2CN; 1 CHC_2N; 3 CH_3C; 1 NHC_2; 1 CH_3N; 1 CH_2C_2 — ΔH = -25.9

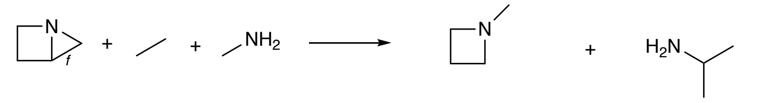

1 NC_3; 2 CH_2CN; 1 CHC_2N; 2 CH_3C; 1 NH_2C; 1 CH_3N; 1 CH_2C_2 — ΔH = -30.5

Figure S16: Balanced hydrogen transfer reactions by bond type, group classifications and reaction enthalpies (kcal mol^{-1}) calculated at the DLPNO-CCSD(T)/def2-QZVPP (TightPNO)//B2PLYP-D3BJ/def2-TZVP level.

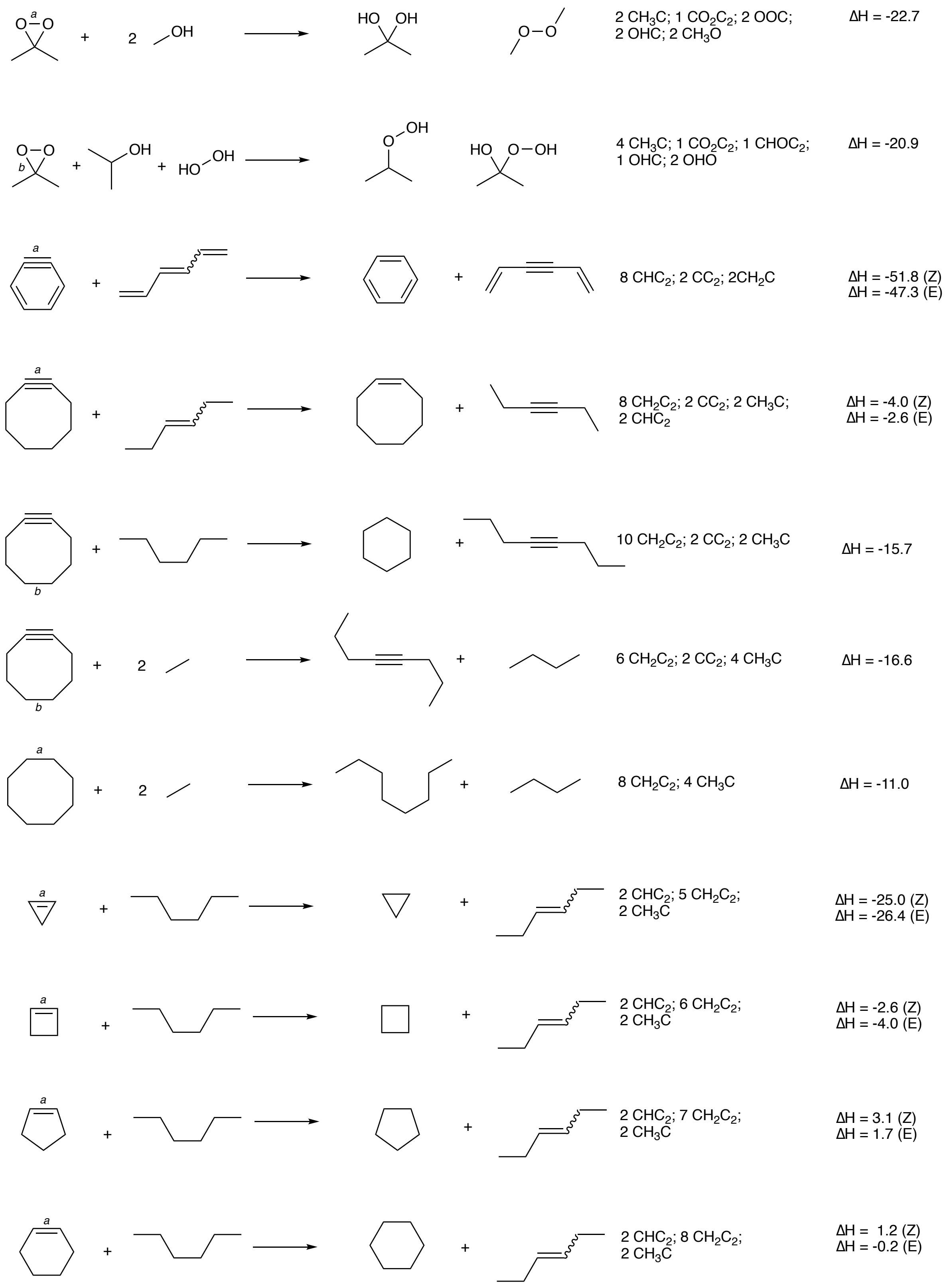

Figure S17: Balanced hydrogen transfer reactions by bond type, group classifications and reaction enthalpies (kcal mol^{-1}) calculated at the DLPNO-CCSD(T)/def2-QZVPP (TightPNO)//B2PLYP-D3BJ/def2-TZVP level.

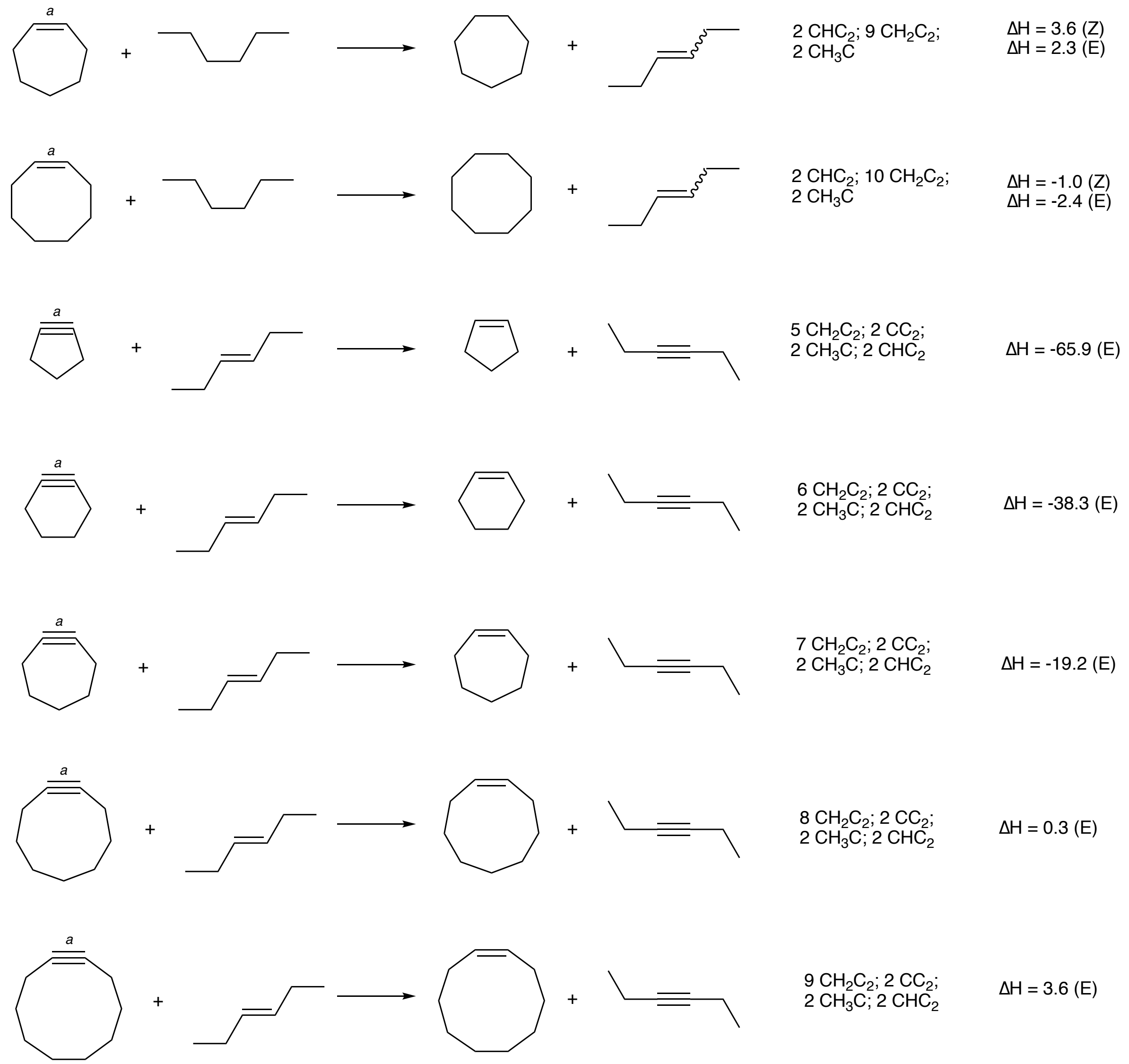

Figure S18: Balanced hydrogen transfer reactions by bond type, group classifications and reaction enthalpies (kcal mol^{-1}) calculated at the DLPNO-CCSD(T)/def2-QZVPP (TightPNO)//B2PLYP-D3BJ/def2-TZVP level.

	Strain release energy (bond 'a') / kcal mol^{-1}			
**Molecule**	**This work**	**Rablen 2020**[21]	**Wiberg 1986**[22]	**L&G 1976**[23]
ortho-Benzyne	-51.8	–	–	-44
Cyclopentyne	-65.9	–	–	–
Cyclohexyne	-38.3	–	–	–
Cycloheptyne	-19.2	–	–	–
Cyclooctyne	-2.6	–	–	–
Cyclononyne	0.3	–	–	–
Cyclodecyne	3.6	–	–	–
Cyclopropene	-26.4	-26.3	-27.7	–
Cyclobutene	-4.0	-3.5	-1.9	–
Cyclopentene	1.7	2.1	2.1	–
Cyclohexene	-0.2	-1.5	0.3	–
Cycloheptene	2.3	–	2.7	–
Cyclooctene	-2.4	–	5.5	–

Table S6: Strain release energies (kcal mol^{-1} for the type 'a' bonds (π) shown in Figures S5–S11, calculated at the DLPNO-CCSD(T)/def2-QZVPP (TightPNO)//B2PLYP-D3bJ/def2-TZVP level (this work). Comparison is made with a variety of values from other sources using different computational methods.[21–23]

	Strain release energy (bond 'a') / kcal mol^{-1}				
**Molecule**	**This work**	**Rablen 2020**[21]	**Wiberg 1986**[22]	**Morgan 2013**[24]	**Expt**
Cyclopropane	-28.3	-27.9	-27.5	–	-27.5^a / -27.6^b
Cyclobutane	-26.4	-26.8	-26.5	–	-26.5^a / -26.2^b
Bicyclo[1.1.0]butane	-40.2	-39.7	-37.4	–	-40.3^b
Bicyclo[2.1.0]pentane	-48.1	-48.1	-48.5	–	-50.8^b
Bicyclo[2.2.0]hexane	-52.5	-52.3	-51.8	–	-50.7^b
Bicyclo[3.1.0]hexane	-34.3	-30.6	-31.0	–	-33.9^b
[1.1.1]Propellane	-28.2	-32.3	-30.0	–	–
[3.1.1]Propellane	-39.2	–	–	–	–
[3.2.1]Propellane	-46.0	-46.0	–	–	–
[2.2.2]Propellane	-78.2	-82.3	-81.6	–	–
Cubane	-48.9	–	–	–	–
Oxirane	-27.6	–	–	-27.1	-27.4^a
Aziridine	-28.1	–	–	-27.5	-27.1^a
Epiphosphine	-21.0	–	–	-19.4	–
Episulfide	-19.0	–	–	-17.6	–
Oxetane	-25.9	–	–	–	–
Azetidine	-26.2	–	–	–	–
Phosphetane	-19.2	–	–	–	–
Thietane	-20.1	–	–	–	–
1-Azabicyclo[1.1.0]butane	-31.4	–	–	–	–
1-Azabicyclo[2.1.0]pentane	-45.3	–	–	–	–
Dimethyldioxirane	-22.7	–	–	–	–

^{a}Taken from ref. [24]. ^{b}Taken from ref. [23].

Table S7: Strain release energies (kcal mol^{-1} for the type 'a' bonds (σ) shown in Figures S5–S11, calculated at the DLPNO-CCSD(T)/def2-QZVPP (TightPNO)//B2PLYP-D3BJ/def2-TZVP level (this work). Comparison is made with a variety of values from other sources using different computational or experimental methods.[21–24]

S7 Azide-alkyne (3+2) cycloaddition reactivity

Alkyne	π NBO 1	π NBO 2	Mean π $2 - N_{occ}$
Hex-3-yne	0.070	0.070	0.070
Cyclooctyne	0.076	0.078	0.077
F_2-Cyclooctyne	0.085	0.091	0.088
Dibenzocyclooctyne	0.088	0.166	0.127
Cycloheptyne	0.078	0.086	0.082
Cylononyne	0.075	0.075	0.075
Cyclodecyne	0.073	0.075	0.074
Monobenzocyclooctyne	0.080	0.124	0.102
Distal monobenzocyclooctyne	0.078	0.085	0.082
N,S-Cyclooctyne	0.077	0.093	0.085

Table S8: delocalization values ($2 - N_{occ}$, in e) for the triple bonds of a selection of alkynes.

Alkyne		ΔE	ΔZPE	ΔH	$T\Delta S$	ΔG
Hex-3-yne	TS	18.3	0.7	18.5	-13.5	32.0
	rxn	-64.7	4.6	-61.5	-15.1	-46.4
Cyclooctyne	TS	9.2	0.6	9.5	-12.8	22.3
	rxn	-76.8	4.9	-73.2	-15.4	-57.8
F_2-Cyclooctyne, syn	TS	5.9	0.8	6.4	-12.9	19.3
	rxn	-78.5	5.2	-74.7	-15.6	-59.1
F_2-Cyclooctyne, anti	TS	8.4	0.7	8.8	-12.7	21.5
	rxn	-76.5	5.0	-72.8	-15.6	-57.2
Dibenzocyclooctyne	TS	5.8	0.9	6.4	-13.4	19.8
	rxn	-70.7	4.5	-67.3	-14.8	-52.5
Cycloheptyne	TS	4.1	0.7	4.5	-12.1	16.6
	rxn	-88.1	5.1	-84.4	-14.9	-69.5
Cyclononyne	TS	15.5	0.5	15.7	-12.5	28.3
	rxn	-68.8	4.7	-65.4	-14.9	-50.5
Cyclodecyne	TS	13.7	0.9	14.1	-13.3	27.4
	rxn	-66.9	5.0	-63.4	-15.7	-47.7
Monobenzocyclooctyne, syn	TS	8.2	0.7	8.6	-12.9	21.4
	rxn	-78.0	5.0	-74.4	-15.5	-58.9
Monobenzocyclooctyne, anti	TS	7.8	0.8	8.2	-13.0	21.2
	rxn	-76.9	4.7	-73.4	-15.1	-58.3
Distal monobenzocyclooctyne	TS	6.4	0.6	6.7	-12.5	19.2
	rxn	-75.2	4.9	-71.6	-14.8	-56.7
N,S-Cyclooctyne, anti	TS	8.8	0.5	9.1	-12.7	21.8
	rxn	-78.6	5.1	-75.0	-15.9	-59.1
N,S-Cyclooctyne, syn	TS	6.5	0.8	6.9	-13.5	20.4
	rxn	-78.8	5.1	-75.3	-16.4	-58.9

Table S9: Differences in thermodynamic quantities (kcal mol^{-1}) for the cycloaddition between methyl azide and a range of alkynes, at the B2PLYP-D3BJ/def2-TZVP level.

S8 References

(1) Neese, F. Software update: the ORCA program system, version 4.0. *Wiley Interdiscip. Rev. Comput. Mol. Sci.* **2017**, *8*, e1327.

(2) Young, T. A.; Silcock, J. J.; Sterling, A. J.; Duarte, F. autodE: Automated Calculation of Reaction Energy Profiles— Application to Organic and Organometallic Reactions. *Angew. Chem. Int. Ed.* **2020**, *60*, 4266–4274.

(3) Riniker, S.; Landrum, G. A. Better Informed Distance Geometry: Using What We Know To Improve Conformation Generation. *J. Chem. Inf. Model.* **2015**, *55*, 2562–2574.

(4) Bannwarth, C.; Ehlert, S.; Grimme, S. GFN2-xTB—An Accurate and Broadly Parametrized Self-Consistent Tight-Binding Quantum Chemical Method with Multipole Electrostatics and Density-Dependent Dispersion Contributions. *J. Chem. Theory Comput.* **2019**, *15*, 1652–1671.

(5) Weigend, F.; Ahlrichs, R. Balanced basis sets of split valence, triple zeta valence and quadruple zeta valence quality for H to Rn: Design and assessment of accuracy. *Phys. Chem. Chem. Phys.* **2005**, *7*, 3297.

(6) Grimme, S. Semiempirical hybrid density functional with perturbative second-order correlation. *J. Chem. Phys.* **2006**, *124*, 034108.

(7) Klopman, G. Solvations: a semi-empirical procedure for including solvation in quantum mechanical calculations of large molecules. *Chem. Phys. Lett.* **1967**, *1*, 200–202.

(8) Barone, V.; Cossi, M. Quantum Calculation of Molecular Energies and Energy Gradients in Solution by a Conductor Solvent Model. *J. Phys. Chem. A* **1998**, *102*, 1995–2001.

(9) Marenich, A. V.; Cramer, C. J.; Truhlar, D. G. Universal Solvation Model Based on Solute Electron Density and on a Continuum Model of the Solvent Defined by the Bulk Dielectric Constant and Atomic Surface Tensions. *J. Phys. Chem. B* **2009**, *113*, 6378–6396.

(10) Riplinger, C.; Neese, F. An efficient and near linear scaling pair natural orbital based local coupled cluster method. *J. Chem. Phys.* **2013**, *138*, 034106.

(11) Neese, F.; Wennmohs, F.; Hansen, A.; Becker, U. Efficient, approximate and parallel Hartree–Fock and hybrid DFT calculations. A 'chain-of-spheres' algorithm for the Hartree–Fock exchange. *Chem. Phys.* **2009**, *356*, 98–109.

(12) Stoychev, G. L.; Auer, A. A.; Neese, F. Automatic Generation of Auxiliary Basis Sets. *J. Chem. Theory Comput.* **2017**, *13*, 554–562.

(13) Grimme, S. Supramolecular Binding Thermodynamics by Dispersion-Correected Density Functional Theory. *Chem. Eur. J.* **2012**, *18*, 9955–9964.

(14) Young, T. duartegroup/otherm: Major symmetry improvements, version 1.0.0 beta, 2020.

(15) Lu, T.; Chen, F. Multiwfn: A multifunctional wavefunction analyzer. *J. Comput. Chem.* **2011**, *33*, 580–592.

(16) Duchesnay, É. Scikit-learn: Machine Learning in Python. *J. Mach. Learn. Res.* **2011**, *12*, 2825–2830.

(17) Hunter, J. D. Matplotlib: A 2D Graphics Environment. *Comput. Sci. Eng.* **2007**, *9*, 90–95.

(18) Becke, A. D.; Edgecombe, K. E. A simple measure of electron localization in atomic and molecular systems. *J. Chem. Phys.* **1990**, *92*, 5397–5403.

(19) Wolk, J. L.; Hoz, T.; Basch, H.; Hoz, S. Quantification of the Various Contributors to Rate Enhancement in Nucleophilic Strain Releasing Reactions. *J. Org. Chem.* **2001**, *66*, 915–918.

(20) Wolk, J. L.; Sprecher, M.; Basch, H.; Hoz, S. Relative reactivity of three and four membered rings – the absence of charge effect. *Org. Biomol. Chem.* **2004**, *2*, 1065.

(21) Rablen, P. R. A Procedure for Computing Hydrocarbon Strain Energies Using Computational Group Equivalents, with Application to 66 Molecules. *Chemistry* **2020**, *2*, 347–360.

(22) Wiberg, K. B.; Waddell, S. T.; Laidig, K. [1.1.1]Propellane: Reaction with free radicals. *Tetrahedron Lett.* **1986**, *27*, 1553–1556.

(23) Liebman, J. F.; Greenberg, A. A survey of strained organic molecules. *Chem. Rev.* **1976**, *76*, 311–365.

(24) Morgan, K. M.; Ellis, J. A.; Lee, J.; Fulton, A.; Wilson, S. L.; Dupart, P. S.; Dastoori, R. Thermochemical Studies of Epoxides and Related Compounds. *J. Org. Chem.* **2013**, *78*, 4303–4311.
