# Supplementary material for: Beyond Strain Release: Delocalization-Enabled Organic Reactivity
Source: J Org Chem. 2024 Jul 6;89(14):9979–89. doi: 10.1021/acs.joc.4c00857 (PMC11267611; doi:10.1021/acs.joc.4c00857)
Supplement: Supplementary file 2 — jo4c00857_si_002.zip [file jo4c00857_si_002.zip › pdfs/Plot4.pdf]

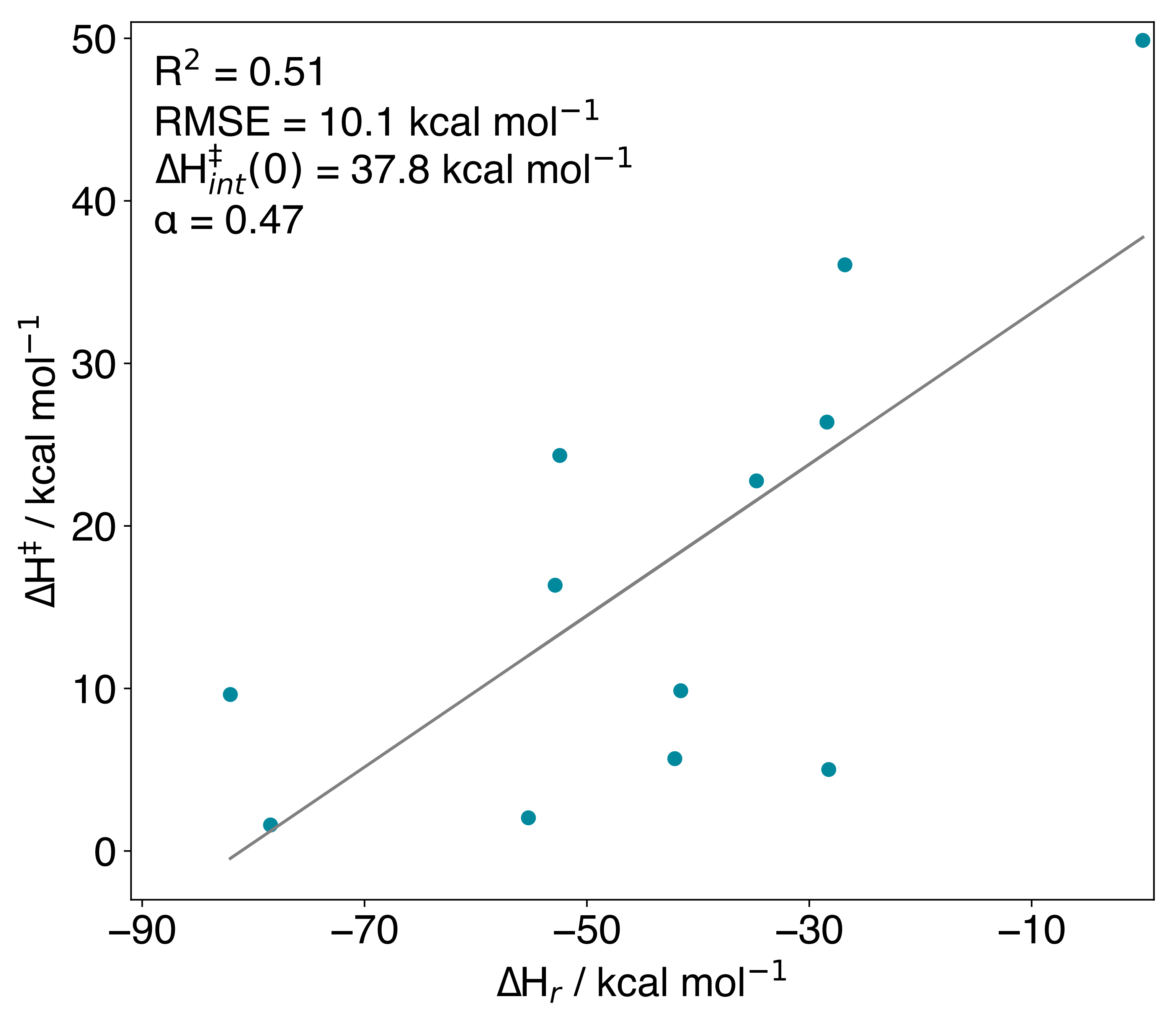

R² = 0.51
RMSE = 10.1 kcal mol⁻¹
ΔH‡int(0) = 37.8 kcal mol⁻¹
α = 0.47
ΔH‡ / kcal mol⁻¹
ΔHr / kcal mol⁻¹
0
10
20
30
40
50
−90
−70
−50
−30
−10
