# Supplementary material for: Beyond Strain Release: Delocalization-Enabled Organic Reactivity
Source: J Org Chem. 2024 Jul 6;89(14):9979–89. doi: 10.1021/acs.joc.4c00857 (PMC11267611; doi:10.1021/acs.joc.4c00857)
Supplement: Supplementary file 2 — jo4c00857_si_002.zip [file jo4c00857_si_002.zip › pdfs/Plot2.pdf]

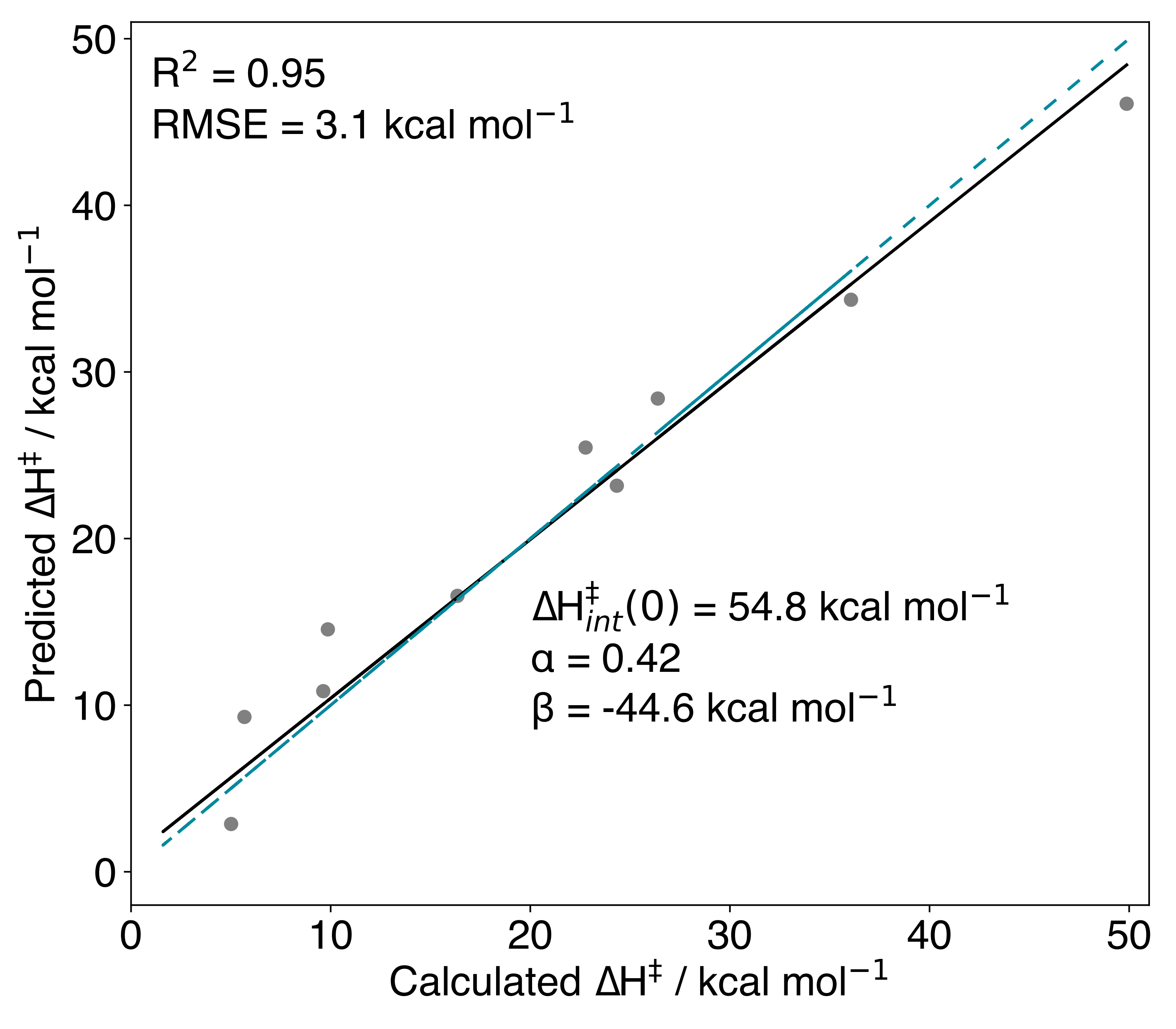

R^2 = 0.95
RMSE = 3.1 kcal mol^{-1}
$\Delta H^{\ddagger}_{int}(0)$ = 54.8 kcal mol^{-1}
α = 0.42
β = -44.6 kcal mol^{-1}
Predicted $\Delta H^{\ddagger}$ / kcal mol^{-1}
Calculated $\Delta H^{\ddagger}$ / kcal mol^{-1}
0
10
20
30
40
50
