# Supplementary material for: Beyond Strain Release: Delocalization-Enabled Organic Reactivity
Source: J Org Chem. 2024 Jul 6;89(14):9979–89. doi: 10.1021/acs.joc.4c00857 (PMC11267611; doi:10.1021/acs.joc.4c00857)
Supplement: Supplementary file 2 — jo4c00857_si_002.zip [file jo4c00857_si_002.zip › pdfs/Plot21.pdf]

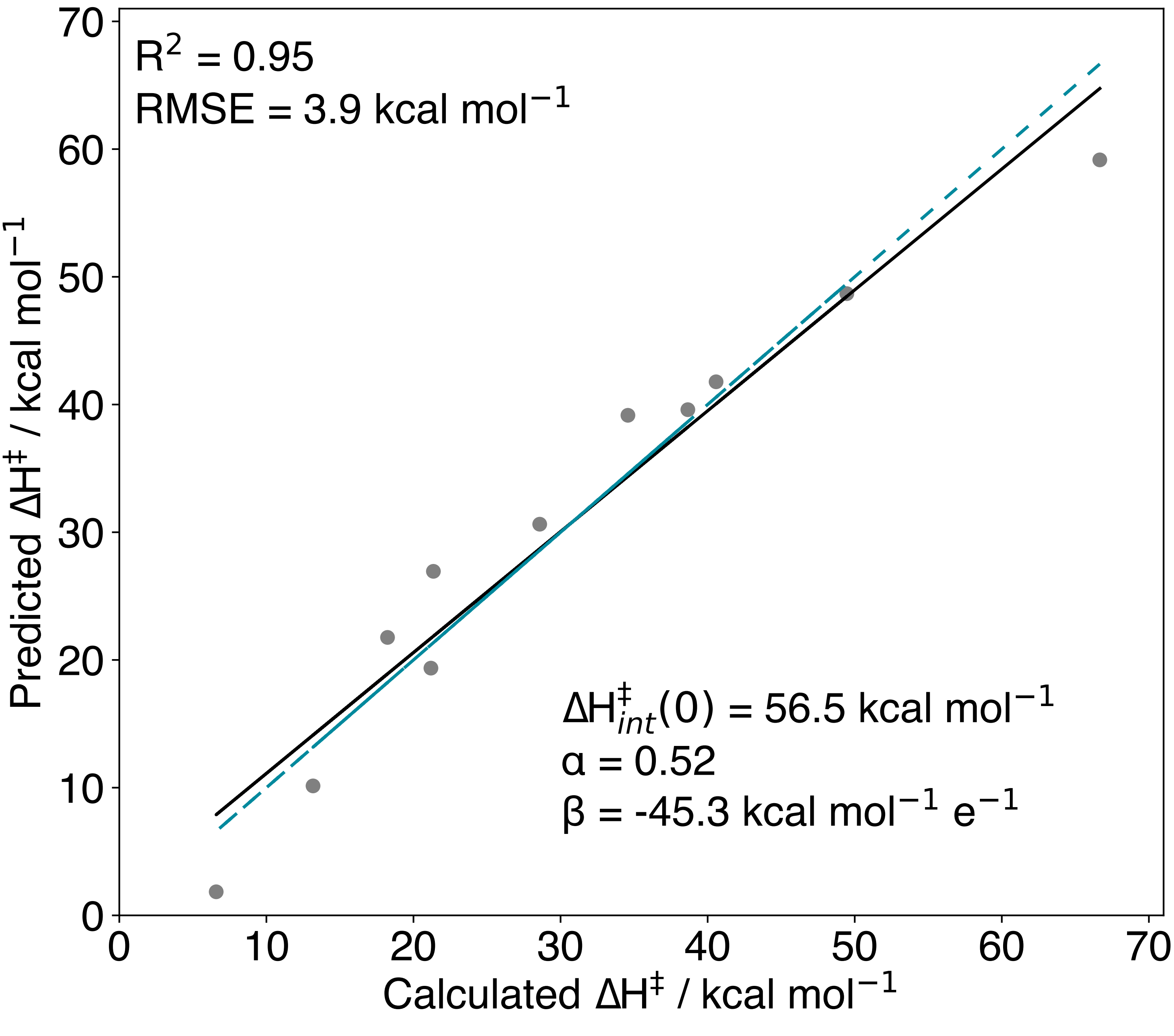

R² = 0.95
RMSE = 3.9 kcal mol⁻¹
ΔH‡int(0) = 56.5 kcal mol⁻¹
α = 0.52
β = -45.3 kcal mol⁻¹ e⁻¹
Predicted ΔH‡ / kcal mol⁻¹
Calculated ΔH‡ / kcal mol⁻¹
0
10
20
30
40
50
60
70
