# Supplementary material for: Beyond Strain Release: Delocalization-Enabled Organic Reactivity
Source: J Org Chem. 2024 Jul 6;89(14):9979–89. doi: 10.1021/acs.joc.4c00857 (PMC11267611; doi:10.1021/acs.joc.4c00857)
Supplement: Supplementary file 2 — jo4c00857_si_002.zip [file jo4c00857_si_002.zip › pdfs/Plot20.pdf]

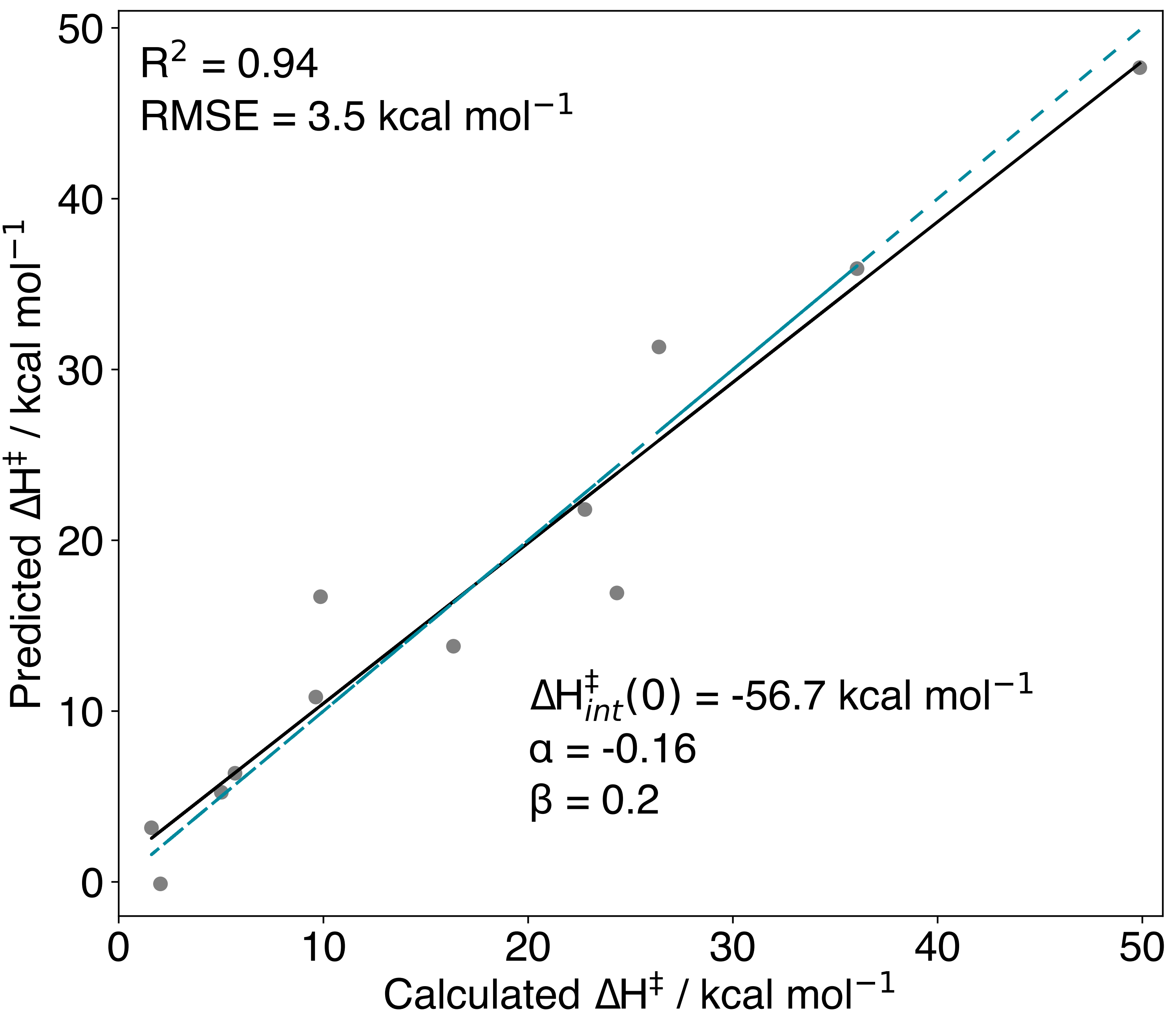

R² = 0.94
RMSE = 3.5 kcal mol⁻¹
ΔH‡int(0) = -56.7 kcal mol⁻¹
α = -0.16
β = 0.2
Predicted ΔH‡ / kcal mol⁻¹
Calculated ΔH‡ / kcal mol⁻¹
0
10
20
30
40
50
