# Supplementary material for: Beyond Strain Release: Delocalization-Enabled Organic Reactivity
Source: J Org Chem. 2024 Jul 6;89(14):9979–89. doi: 10.1021/acs.joc.4c00857 (PMC11267611; doi:10.1021/acs.joc.4c00857)
Supplement: Supplementary file 2 — jo4c00857_si_002.zip [file jo4c00857_si_002.zip › pdfs/Plot10.pdf]

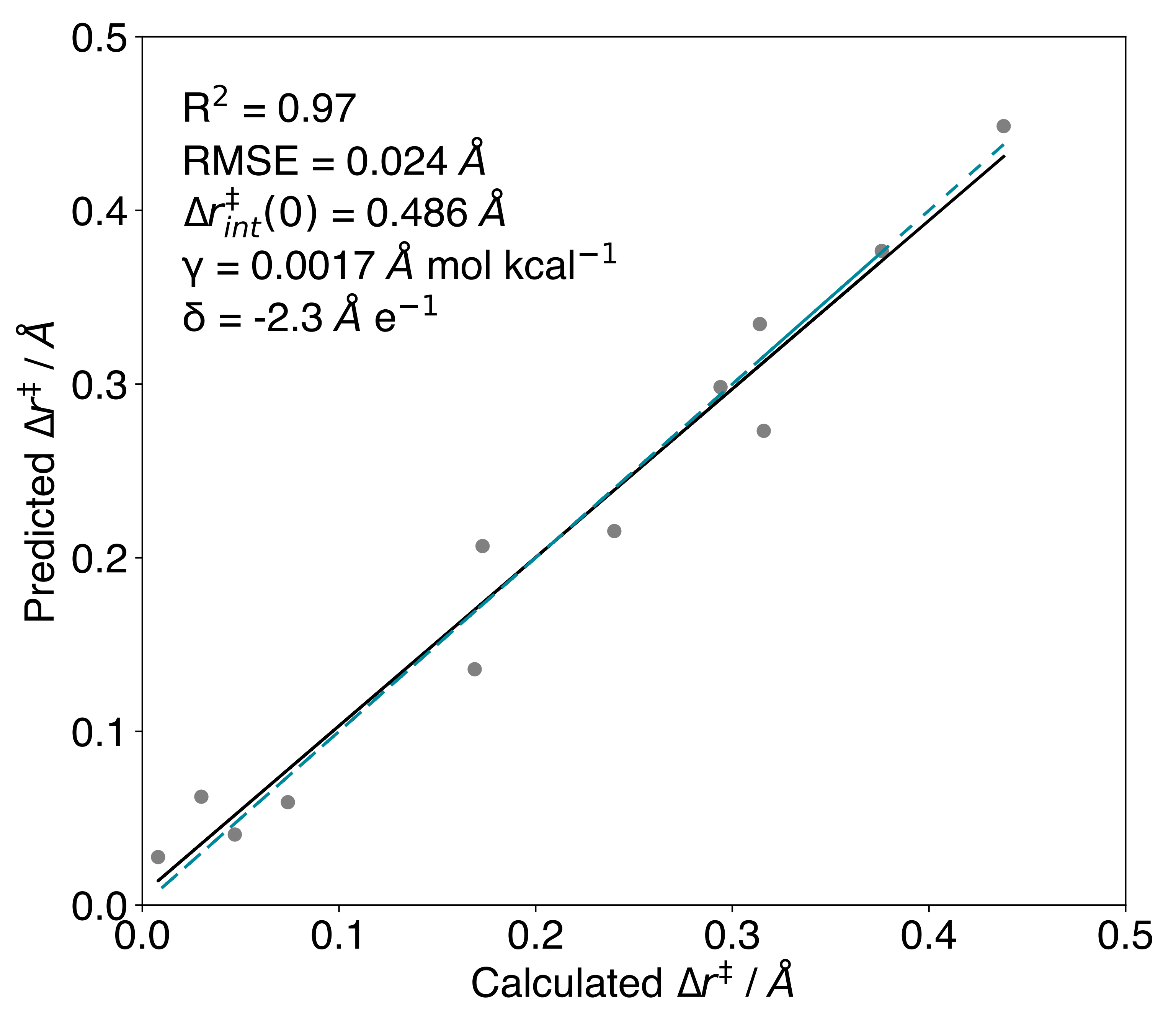
R^2 = 0.97
RMSE = 0.024 Å
$\Delta r^{\ddagger}_{int}(0)$ = 0.486 Å
γ = 0.0017 Å mol $kcal^{-1}$
δ = -2.3 Å e^{-1}
Predicted $\Delta r^{\ddagger}$ / Å
Calculated $\Delta r^{\ddagger}$ / Å
0.0
0.1
0.2
0.3
0.4
0.5
