# Supplementary material for: Beyond Strain Release: Delocalization-Enabled Organic Reactivity
Source: J Org Chem. 2024 Jul 6;89(14):9979–89. doi: 10.1021/acs.joc.4c00857 (PMC11267611; doi:10.1021/acs.joc.4c00857)
Supplement: Supplementary file 2 — jo4c00857_si_002.zip [file jo4c00857_si_002.zip › pdfs/Plot13.pdf]

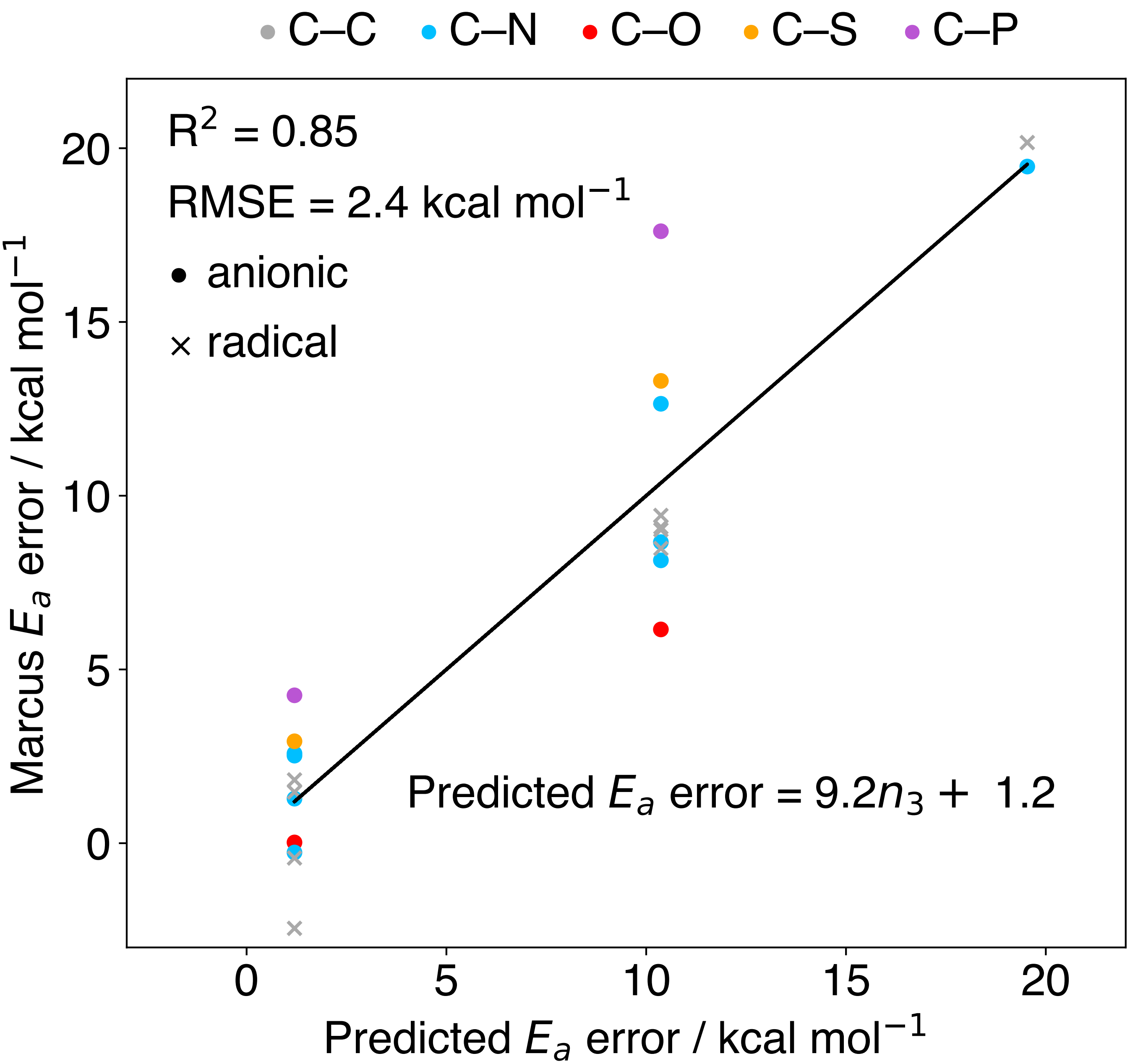
C–C
C–N
C–O
C–S
C–P
R2 = 0.85
RMSE = 2.4 kcal mol−1
anionic
radical
Predicted Ea error = 9.2n3 + 1.2
Marcus Ea error / kcal mol−1
Predicted Ea error / kcal mol−1
0
5
10
15
20
