# Supplementary material for: Beyond Strain Release: Delocalization-Enabled Organic Reactivity
Source: J Org Chem. 2024 Jul 6;89(14):9979–89. doi: 10.1021/acs.joc.4c00857 (PMC11267611; doi:10.1021/acs.joc.4c00857)
Supplement: Supplementary file 2 — jo4c00857_si_002.zip [file jo4c00857_si_002.zip › pdfs/Plot17.pdf]

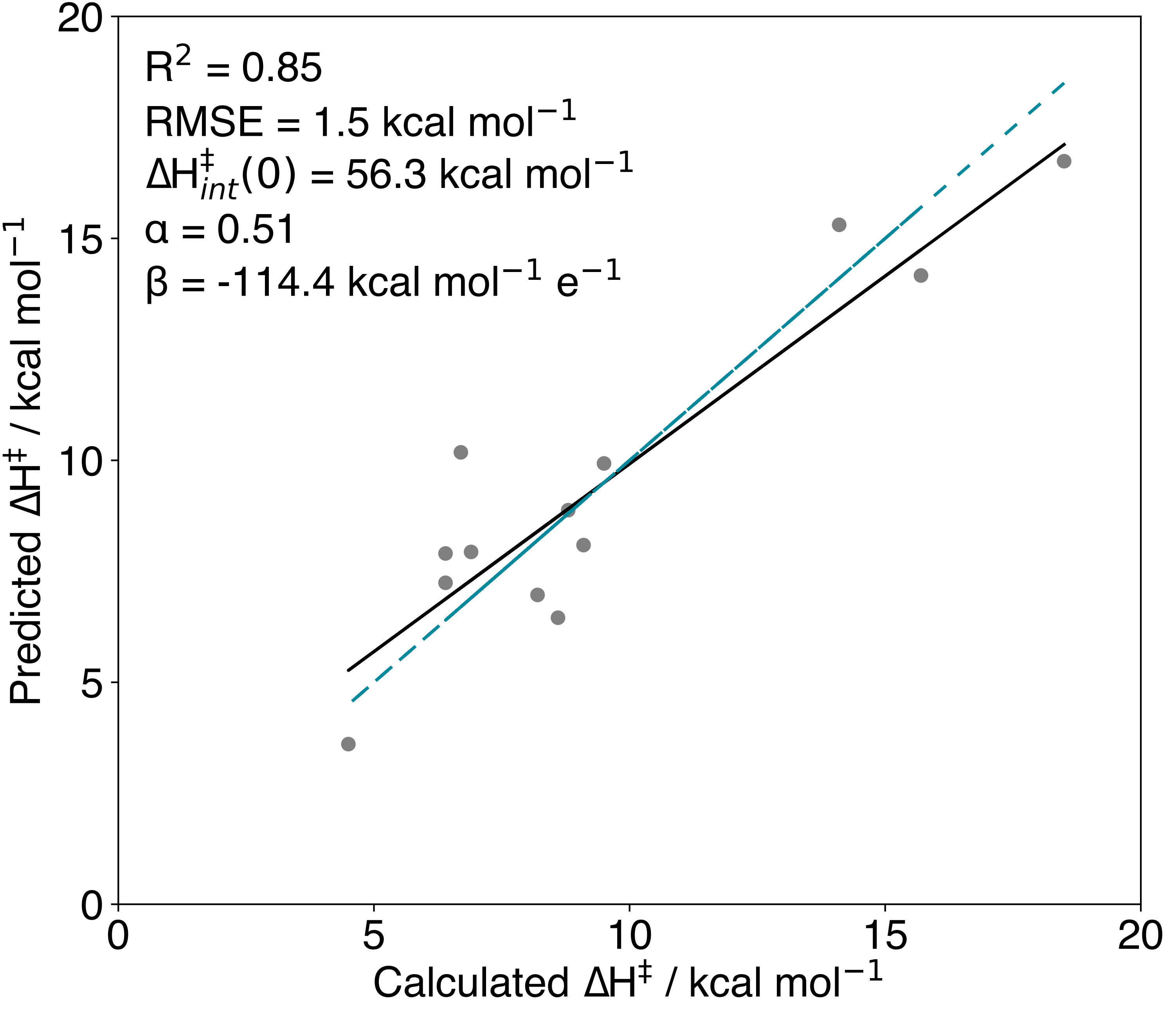

R2 = 0.85
RMSE = 1.5 kcal mol−1
ΔH‡int(0) = 56.3 kcal mol−1
α = 0.51
β = -114.4 kcal mol−1 e−1
Predicted ΔH‡ / kcal mol−1
Calculated ΔH‡ / kcal mol−1
0
5
10
15
20
