# Supplementary material for: Beyond Strain Release: Delocalization-Enabled Organic Reactivity
Source: J Org Chem. 2024 Jul 6;89(14):9979–89. doi: 10.1021/acs.joc.4c00857 (PMC11267611; doi:10.1021/acs.joc.4c00857)
Supplement: Supplementary file 2 — jo4c00857_si_002.zip [file jo4c00857_si_002.zip › pdfs/Plot9.pdf]

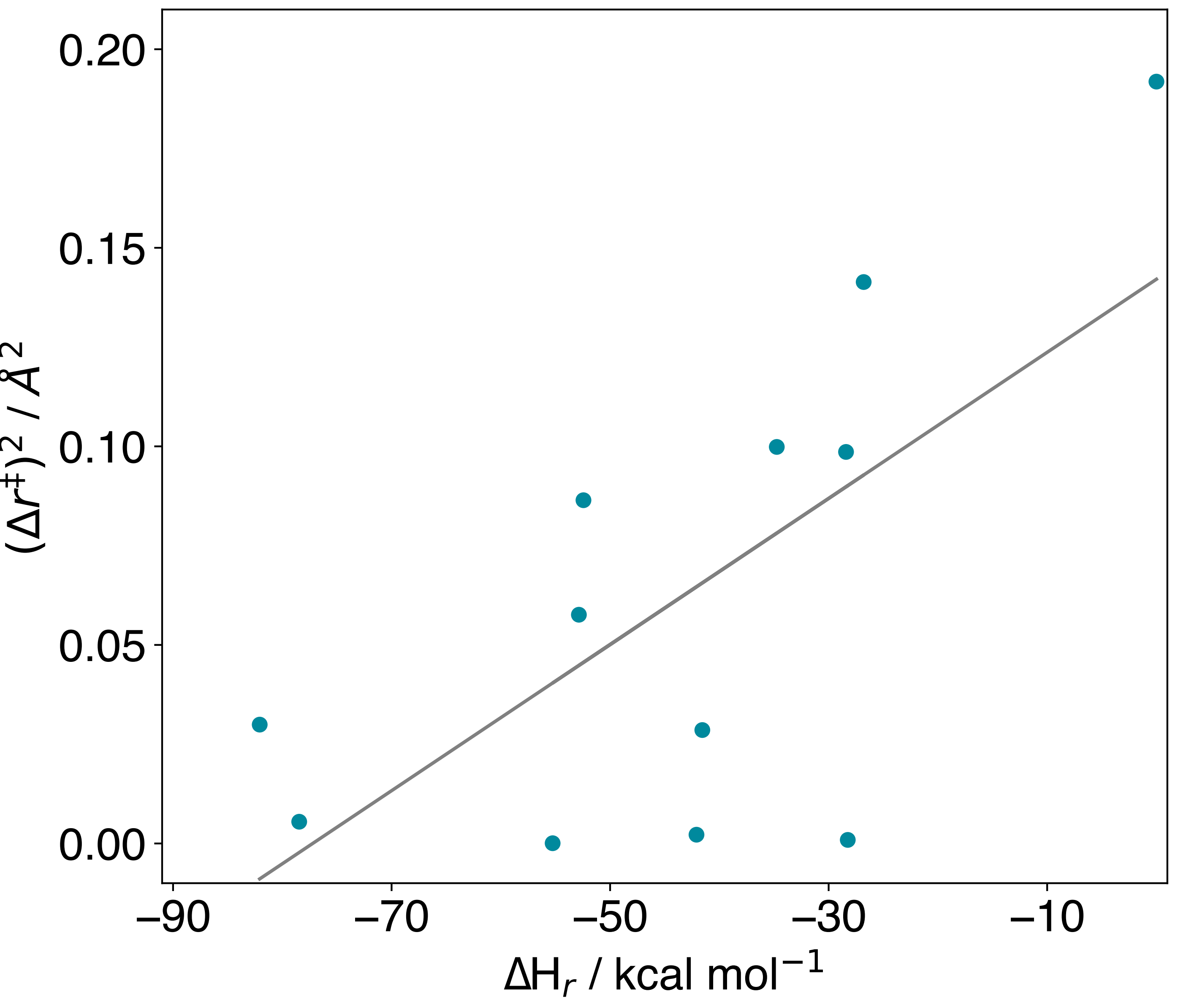

0.20
0.15
0.10
0.05
0.00
−90
−70
−50
−30
−10
$(\Delta r^{\ddagger})^2$ / Å^2
ΔH_r / kcal mol^{-1}
