# Supplementary material for: Beyond Strain Release: Delocalization-Enabled Organic Reactivity
Source: J Org Chem. 2024 Jul 6;89(14):9979–89. doi: 10.1021/acs.joc.4c00857 (PMC11267611; doi:10.1021/acs.joc.4c00857)
Supplement: Supplementary file 2 — jo4c00857_si_002.zip [file jo4c00857_si_002.zip › pdfs/Plot8.pdf]

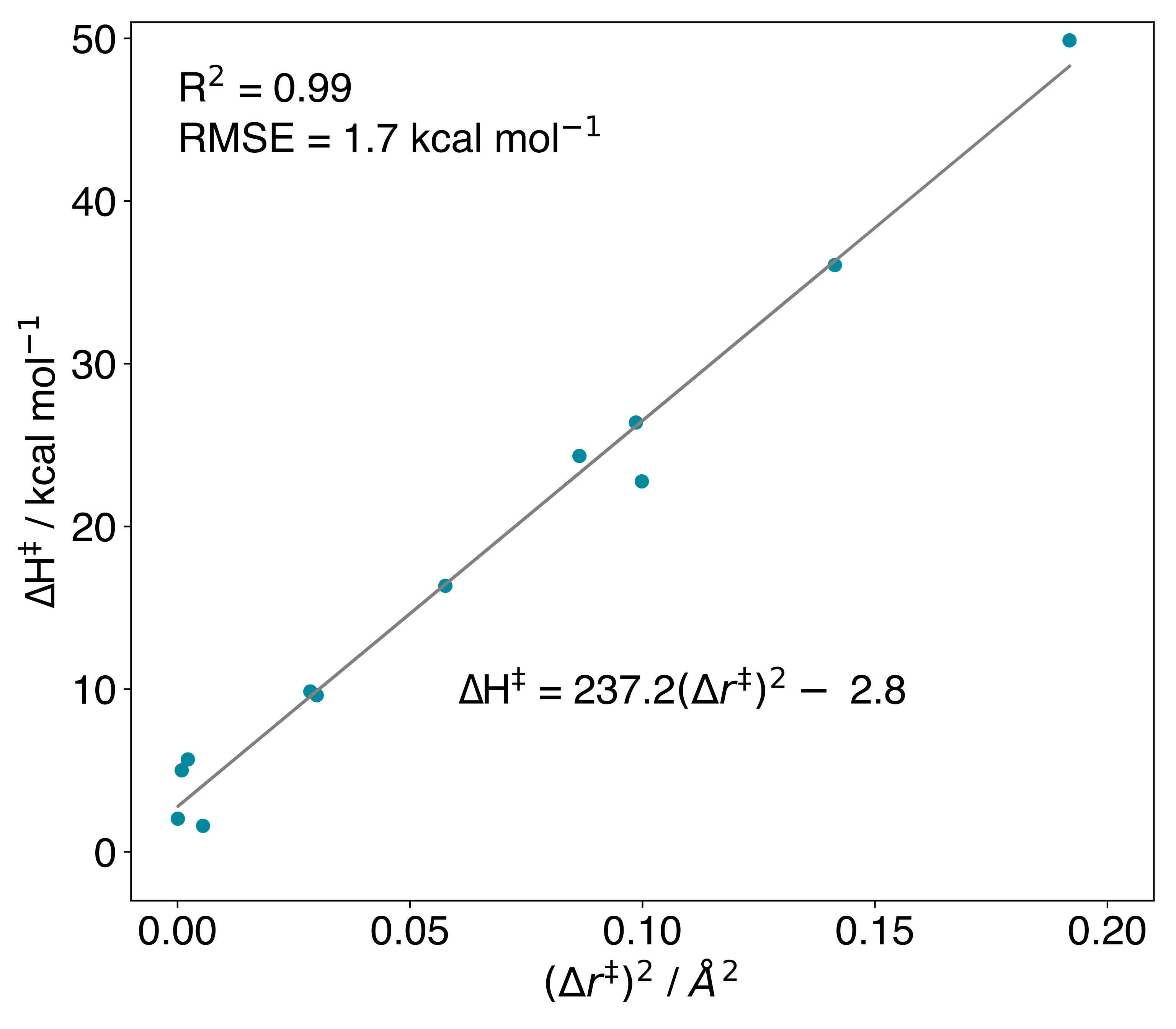

$R^2 = 0.99$
RMSE = 1.7 kcal mol^{-1}
$\Delta H^{\ddagger} = 237.2(\Delta r^{\ddagger})^2 - 2.8$
$\Delta H^{\ddagger}$ / kcal mol^{-1}
$(\Delta r^{\ddagger})^2$ / $Å^2$
0
10
20
30
40
50
0.00
0.05
0.10
0.15
0.20
